# Hydrogel-Based Continuum Soft Robots

**DOI:** 10.3390/gels11040254

**Published:** 2025-03-27

**Authors:** Honghong Wang, Jingli Du, Yi Mao

**Affiliations:** 1 School of Mechano-Electronic Engineering, Xidian University, Xi’an 710071, China; 2 School of Chemical and Material Engineering, Jiangnan University, Wuxi 214122, China; maoyi@stu.jiangnan.edu.cn

**Keywords:** hydrogel, soft robots, continuum robot, continuum soft robots, hydrogel-based

## Abstract

This paper comprehensively reviews the latest advances in hydrogel-based continuum soft robots. Hydrogels exhibit exceptional flexibility and adaptability compared to traditional robots reliant on rigid structures, making them ideal as biomimetic robotic skins and platforms for constructing highly accurate, real-time responsive sensory interfaces. The article systematically summarizes recent research developments across several key dimensions, including application domains, fabrication methods, actuator technologies, and sensing mechanisms. From an application perspective, developments span healthcare, manufacturing, and agriculture. Regarding fabrication techniques, the paper extensively explores crosslinking methods, additive manufacturing, microfluidics, and other related processes. Additionally, the article categorizes and thoroughly discusses various hydrogel-based actuators responsive to solute/solvent variations, pH, chemical reactions, temperature, light, magnetic fields, electric fields, hydraulic/electro-osmotic stimuli, and humidity. It also details the strategies for designing and implementing diverse sensors, including strain, pressure, humidity, conductive, magnetic, thermal, gas, optical, and multimodal sensors. Finally, the paper offers an in-depth discussion of the prospective applications of hydrogel-based continuum soft robots, particularly emphasizing their potential in medical and industrial fields. Concluding remarks include a forward-looking outlook highlighting future challenges and promising research directions.

## 1. Introduction

Robotics has a long and diverse developmental history. It is generally accepted that modern robotics began with the programmable industrial robot Unimate in the 1950s. The concept of redundant robots emerged shortly thereafter in the 1960s [1], followed by the advent of hyper-redundant robots marked by the development of a snake-like robot with 20 degrees of freedom in 1972 [2]. By the end of the 20th century, the concept of continuum robots was also proposed [3,4]. Although soft robots were introduced following rigid robotics [5], their initial advancement was limited by constraints in materials and actuation technologies available at the time. However, entering the 21st century, soft robotics experienced unprecedented growth and vitality, primarily driven by integrating advanced manufacturing technologies [6]. This leap forward can largely be attributed to the flexibility of silicone materials and the synergistic integration of various actuation methods, including fluid-driven [7,8], electric-driven [9], tendon-driven [10,11], and magnetic-driven approaches [12,13]. As application domains have continued to expand, limitations of silicone materials, particularly regarding biocompatibility and environmental adaptability, have become increasingly evident, prompting researchers to investigate alternative flexible materials. Concurrently, hydrogels, known for their excellent responsiveness and biocompatibility, have attracted significant interest across materials science, environmental science, biochemistry, and medicine [14,15,16]. This new class of materials quickly gained attention from researchers specializing in soft robotics and hydrogel materials [17,18]. Leveraging extensive research on hydrogels, the concept of hydrogel-based continuum soft robotics was rapidly developed [19,20,21].

Early research and applications of hydrogels primarily focused on biomedical fields [22,23,24]. As the unique physical properties of hydrogels became increasingly recognized [25,26,27], researchers began to explore their potential as smart materials [15,28], particularly their responsiveness to various stimuli, such as temperature [29], pH [30], electric fields [31], and magnetic fields [32,33]. These advances laid the foundation for the development of hydrogel-based continuum soft robots. The latest generation of hydrogels aims at integrating multiple intelligent functions into the materials to enable diverse capabilities in hydrogel-based continuum soft robots, including intelligent drug delivery [34,35], adaptive shape changes for navigation through constrained spaces [36,37], and underwater robotics applications [38,39]. In recent years, hydrogel technologies have also made significant progress in environmental protection and circular economy. For instance, Heiden et al. [40] utilized industrial waste materials as raw resources to fabricate hydrogel-based continuum soft robots. Moreover, hydrogel-based continuum soft robots demonstrate substantial potential for integrating sensing and actuation into unified, functional platforms [41,42,43], showing particular promise in biomedical applications such as minimally invasive surgery and targeted drug delivery [44,45], as well as contributing to sustainable development through recycling industrial waste and enhancing environmental protection in agriculture [46,47]. This approach expands hydrogels’ functionality and application scope and provides novel perspectives for environmental protection and water purification strategies [48,49,50].

Since the 1960s, hydrogel materials have evolved progressively from single-function materials to functional materials and subsequently into multifunctional integrated systems, ultimately enabling the construction of hydrogel-based continuum soft robots. Although such robotic systems remain at a preliminary developmental stage, hydrogels’ exceptional softness, adaptability, and multimodal responsiveness indicate significant potential and promising application value in future robotic technologies. Existing humanoid robots and bio-inspired robotic dogs predominantly rely on rigid structures, which inherently pose safety risks in human–robot interactions, significantly when system programs or structural designs are compromised. In contrast, hydrogels are ideal for robotic skin or sensory interfaces, enabling enhanced biomimetic fidelity and safer human–robot interaction. To advance the development and application of hydrogels within the field of soft robotics, this paper systematically reviews the relevant literature. Given that previous studies [19,20] have comprehensively summarized research conducted before 2020, the present review primarily focuses on the most recent developments from 2020 to 2025.

From a robotics perspective, this review first provides an overview of various applications of hydrogel-based continuum soft robots. Then, it introduces, in detail, the structural fabrication methods, responsive actuators, and sensors. The remainder of this paper is structured into six sections: Section 2 focuses on the applications of hydrogel-based continuum soft robots, primarily covering industrial, medical, and agricultural fields; Section 3 describes the fabrication methods, including crosslinking techniques, additive manufacturing, and microfluidic technologies; Section 4 discusses actuators, encompassing solute- or solvent-responsive, pH-responsive, chemically responsive, temperature-responsive, photo-responsive, magnetically responsive, electrically responsive, hydraulically or electro-osmotically responsive, humidity-responsive, and multifunctional actuators; Section 5 systematically classifies sensors, specifically addressing strain, pressure, humidity-sensitive, conductive, magnetically sensitive, temperature-sensitive, gas-sensitive, photosensitive, and multimodal hydrogel-based sensors; Section 6 presents the discussion; and Section 7 provides the conclusions.

## 2. Applications of Hydrogel-Based Continuum Soft Robots

### 2.1. Industrial Applications

With the continuous advancements in flexible materials and intelligent control technologies, hydrogel-based continuum soft robots—as a novel class of flexible robots—are gradually attracting widespread attention in the industrial sector [19,51,52] (Table 1). Gao et al. [53] developed an ionic hydrogel sensor that integrates collagen with polyacrylamide, enabling efficient monitoring of electrocardiograms and human motion while demonstrating exceptional performance even under extreme temperature conditions. Guan et al. [54] significantly enhanced the mechanical properties and ionic stimulus responsiveness of metal-coordinated hydrogels for applications in flexible electronics. Xu et al. [55] developed a loofah-inspired solar-absorbing gel capable of rapidly extracting potable water from various contaminant sources and effectively purifying pollutants such as oils, metals, and microplastics. Wang et al. [56] introduced a multifunctional ionic hydrogel that maintains rapid self-healing, ultra-high stretchability, and stable electrical conductivity even at extremely low temperatures, thereby demonstrating the tremendous potential for bionic intelligent robots operating in harsh environments. Yu et al. [57] focused on exploring the classification of hydrogels in environmental applications, the crosslinking methods employed, and the mechanisms underlying pollutant adsorption. Liu et al. [58] developed a novel multifunctional self-sensing gradient hydrogel that, owing to its ultra-fast thermoresponsive actuation and high sensitivity, provides robust support for the realization of interactive robotic interfaces. Hsiao et al. [59] integrated carbon nanomaterials into hydrogels and successfully developed a robotic skin exhibiting high deformability and multiple embedded functionalities. In contrast, Banerjee et al. [60] proposed a fire-resistant robotic scheme employing montmorillonite-based biocompatible hydrogel skin, suitable for various applications, including firefighting and the packaging of energy storage devices. Furthermore, Wen et al. [61] reported an enhanced three-dimensional data encryption security technique based on biodegradable pH-responsive hydrogels, which manipulates information through pH variation; in a subsequent study, a corresponding decryption method was also proposed [62].

### 2.2. Medical Applications

**Figure 1:**
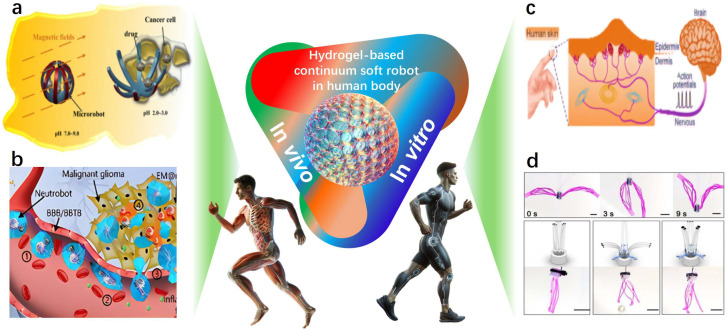
Hydrogel-based continuum soft robots in vitro and in vivo: (**a**) magnetically controlled targeted drug-delivery microrobots (reprinted with permission from [70]); (**b**) micro- and nano-scale drug-delivery robots (reprinted with permission from [72]); (**c**) skin-inspired hydrogel-based sensors (reprinted with permission from [83]); and (**d**) spinning-designed weaveable hydrogel fiber robots (reprinted with permission from [91]).

Medical applications of hydrogel-based continuum soft robots rapidly expand in diverse forms, demonstrating the significant achievements attained through in-depth research. These applications encompass wound healing [63,64,65], functional antimicrobial strategies [66], and various specialized therapeutic approaches [14,67]. Specifically, hydrogels have shown versatility and innovation as interface materials for both in vivo and in vitro applications (Figure 1 and Table 2). For targeted disease therapies, Liu et al. [68] developed a multifunctional nanocomposite hydrogel that provides novel strategies for addressing oxidative stress in retinal injury models. Additionally, ultrasound-responsive hydrogels facilitate stem cell homeostasis and differentiation during bone defect repair through selenoprotein-mediated antioxidant activity [69]. Furthermore, Li et al. [70] proposed a dual-control hydrogel-based continuous soft robot designed for targeted transport and intelligent release of anticancer drugs (Figure 1a). Similarly, the assembly technology of hydrogel micro-units based on sodium alginate and chitosan has been applied in the magnetically anisotropic programming of microrobots [71]. The micro- and nanoscale soft robots utilizing gelatin nanogels have also demonstrated significant potential in actively delivering drugs to malignant gliomas [72] (Figure 1b), underscoring hydrogel’s considerable promise in precision medicine and targeted therapy. Regarding biomedical devices and materials, Liu et al. [73] reported using polyvinyl alcohol (PVA) hydrogels in artificial biological soft tissues and implantable devices. Tian et al. [74] utilized radiation-induced infiltration polymerization to convert traditional elastomers into hybrid hydrogels with both hydrophobic networks and hydrophilic chains. These hydrogels exhibit mechanical properties similar to human skin, excellent compressive and puncture resistance, and shape adaptability. Chen et al. [75] introduced a biodegradable magnetic hydrogel robot capable of flexible switching among four stable locomotion modes through a self-developed visual-guided magnetic actuation system, thus achieving targeted drug delivery.

In terms of in vitro applications, hydrogels have demonstrated extensive applicability across multiple diagnostic and therapeutic innovation domains. Electroadhesive hydrogel interfaces, activated electrically, achieve strong and long-lasting mucosal adhesion, thereby providing novel solutions for diagnosing and treating gastrointestinal and other related diseases [77]. Additionally, the temperature-sensitive adhesive hydrogel patch developed by Jiang et al. [76] introduces a novel approach to painless wound treatment, particularly beneficial for premature infants and diabetic patients. Hydrogels also exhibit diversified applications in medical assistance and drug delivery systems. For example, their use in simulating robotic joints and muscles has been explored [18], along with applications in cancer therapy [78], diabetes treatment [79,84], intraoral wound dressings [80,85], and rapid surgical hemostasis and wound sealing [81]. Concurrently, hydrogels play a critical role in the development of wearable devices: skin-like hydrogel sensors provide efficient design solutions for tendon-driven continuum soft robots (Figure 1c) [83], while gelatin-based hydrogels offer innovative, durable, and biodegradable materials for soft actuators and autonomous electronic platforms [86]. Moreover, superelastic conductive hydrogels provide soft robots with exceptional sensing capabilities and satisfy energy self-sufficiency requirements [87]. In innovations about sensors and intelligent materials, high-performance, environmentally friendly ionic hydrogels have been integrated into strain-triboelectric sensors, demonstrating the significant potential for underwater intelligent, flexible grasping, and wearable sensing applications [88]. Furthermore, the biomimetic ionic gel skin and two-dimensional material-based hydrogel skins employed in continuum robots [89,90], along with hydrogel-based continuum soft robots [91] (Figure 1d), further highlight the innovative accomplishments and extensive application prospects within this field.

### 2.3. Agriculture and Other Applications

Hydrogel-based continuum soft robots are increasingly significant in modern agriculture and food sciences, demonstrating broad application potential [92,93,94] (Table 3). In agricultural applications, Liu et al. [95] utilized highly absorbent hydrogels to achieve controlled fertilizer release and water management. Meanwhile, Yan et al. [96] developed plant-wearable hydrogel discs, offering a novel solution for the highly sensitive detection of pesticide residues. Furthermore, Palanivelu et al. [97] discussed the applications of hydrogels in urban agriculture. Li et al. [98] explored the potential agricultural uses of biopolymer-based hydrogels designed for atmospheric water harvesting. Kaur et al. [99] demonstrated improvements in crop drought resistance, key nutrient storage, seed coating efficiency, and transplant success rates using hydrogels. Xu et al. [100] reported using hymenal-triggered polysaccharide supramolecular hydrogels to enhance soil water retention and control fungicide release. Moreover, lignin-based hydrogels synthesized by Adjuik et al. [101] exhibited superior performance in improving the water retention capabilities of sandy and silty soils. Additionally, poly(N-isopropyl acrylamide) hydrogels successfully prepared by Tamer et al. [102] were employed for storing nitrate fertilizers. In the field of food science, hydrogel-based continuum soft robots have been extensively utilized for bioactive compound delivery [103], active compound release systems [104], and food safety monitoring [105,106]. These applications also provide innovative strategies for intelligent food preservation [107,108,109,110]. In cosmetic applications, such robots not only facilitate skin disease treatment [111] and intelligent skin hydration [112] but also delay skin aging [113] and enable intelligent ultraviolet protection [114].

Despite notable advancements in hydrogel-based continuum soft robotics across diverse sectors such as industrial automation, medical applications, agriculture, food sciences, and cosmetics, significant challenges persist in transitioning these innovations from laboratory demonstrations to scalable, real-world applications. Although these robots demonstrate promising characteristics—including environmental resilience, enhanced biocompatibility, multifunctional sensing, and stimulus responsiveness—their long-term operational stability, durability under multi-factorial environments, and comprehensive economic feasibility remain insufficiently explored. For instance, industrial applications often confront manufacturing processes and reliability complexities under combined environmental stresses, hindering scalable deployment. Despite remarkable progress in targeted therapies, wearable sensing, and bio-mimetic devices in the medical sector, substantial gaps remain regarding biodegradation control, reproducible dosage accuracy, extended biocompatibility, immune response management, and reliable clinical performance validation. Similarly, agricultural and food science applications face uncertainties regarding hydrogels’ scalability, sustainability, regulatory compliance, and ecological impacts under realistic large-scale conditions. At the same time, cosmetic implementations necessitate rigorous in vivo evaluations to confirm safety and efficacy under varied physiological scenarios. Therefore, future research should prioritize systematic validation through extensive real-world and clinical trials, streamlined manufacturing protocols, thorough sustainability assessments, and detailed cost–benefit analyses to facilitate robust, reliable, and economically viable hydrogel-based continuum soft robotics deployment across multiple practical domains.

## 3. Fabrication of Hydrogel-Based Continuum Soft Robots

### 3.1. Crosslinking Method

Regarding hydrogel-based continuum soft robots, crosslinking methods are primarily categorized into chemical and physical crosslinking. These methods’ underlying principles and strategies provide robust support for achieving exceptional mechanical properties and superior biocompatibility. Chemical crosslinking predominantly employs chemical techniques such as radical polymerization and condensation reactions to construct crosslinked networks, which apply to a wide range of synthetic and natural polymer materials. In contrast, physical crosslinking utilizes mild processes, including freeze–thaw cycles, ionic crosslinking, and molecular self-assembly, making it particularly suitable for applications requiring high chemical stability and biocompatibility (Table 4). Wang et al. [122] provided an extensive review of novel hydrogel systems based on polymeric monomers, elaborating on various chemical and physical crosslinking strategies and their influence on hydrogel responsiveness to environmental stimuli. Dodda et al. [123] examined recent trends in multi-component hydrogel crosslinking within the biomedical field, emphasizing multi-component systems’ complexity and potential applications. Huang et al. [124] systematically summarized advances in double-network hydrogels, detailing the strengths and weaknesses of multiple crosslinking techniques and their contributions to improved mechanical properties. Furthermore, Nasution et al. [125] focused on crosslinking mechanisms in cellulose-based hydrogels, systematically comparing the effects of various crosslinkers on their physicochemical properties and performance. Gao et al. [126] explored covalently crosslinked hydrogels’ chemical characteristics and clinical translation potential, demonstrating their importance in biomedical applications. Meanwhile, Ma et al. [127] and Stubbe et al. [128] delved deeply into the mechanisms and advantages of photo-crosslinked hydrogels for wound healing, highlighting significant clinical potential.

Moreover, various studies complement one another, highlighting the diverse applications and innovations associated with hydrogel crosslinking technology. Zhang et al. [145] and Gholami et al. [146] demonstrated the therapeutic efficacy of injectable photo-crosslinked hydrogels for infected wound treatment and injectable thermo-responsive hydrogels for hepatocyte therapy, respectively, underscoring the potential of hydrogel in medical applications. Rizwan et al. [147] and Jaramillo et al. [148] investigated dynamic covalent crosslinking hydrogels and sericin-carboxymethyl cellulose-polyvinyl alcohol composite hydrogels, emphasizing their significance in tissue engineering and 3D cell culture applications. Additionally, Salma et al. [149], and Erikci et al. [150] analyzed the effects of different crosslinking strategies on antibacterial properties and environmental responsiveness, illustrating innovative approaches to adapting hydrogels to specific environmental conditions. Simultaneously, Xu et al. [151], Kkedzierska et al. [152], and Bao et al. [153] thoroughly discussed rapid dual crosslinking strategies, the influence of crosslinkers on physicochemical properties, and the relationships between crosslinked structures, mechanical performance, and microstructure, laying a theoretical and practical foundation for precise hydrogel design in wound dressings and materials science. Finally, Debertrand et al. [154] conducted in-depth research on the mechanical performance of dual-crosslinked hydrogels, identifying specific pathways to optimize hydrogel performance through crosslinking techniques. Chen et al. [155] introduced a novel microwave-assisted drying method for fabricating crosslinked microneedle arrays, significantly reducing preparation time while maintaining material stability and consistency (additional crosslinking methods are listed in Table 4).

### 3.2. Additive Manufacturing

**Figure 2:**
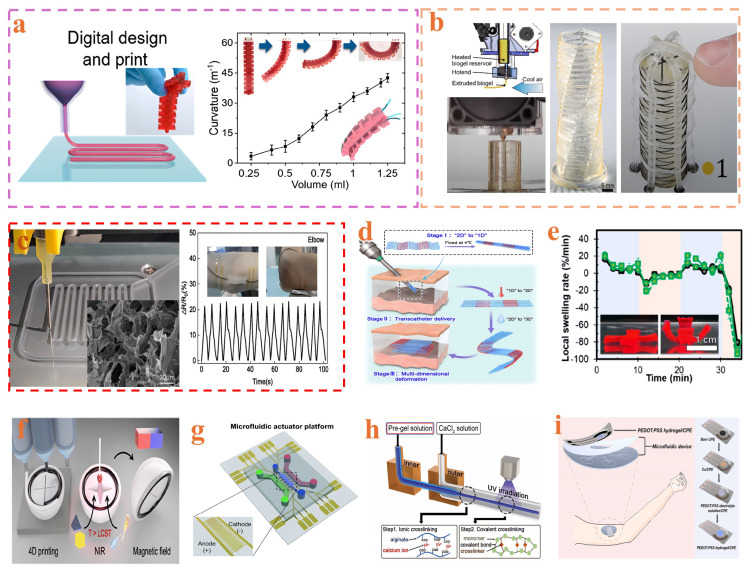
Additive manufacturing and microfluidics: (**a**) schematic illustration of 3D-printed hydrogel soft robots exhibiting unidirectional bending (reprinted with permission from [161]); (**b**) elastic biogel fabricated through 3D printing (reprinted with permission from [40]); (**c**) wearable sensors based on 3D-printed hydrogels (reprinted with permission from [159]); (**d**) multidimensional deformation from 1D to 3D of temperature- and water-responsive scaffolds fabricated via 4D printing for minimally invasive implantation (reprinted with permission from [166]); (**e**) shape evolution of 4D-printed hydrogels over time under external stimuli (reprinted with permission from [170]); (**f**) smart soft carriers with dual-stimulus responsiveness fabricated by multimaterial 4D printing (reprinted with permission from [175]); (**g**) electro-responsive hydrogel microfluidic actuator platform applied to photothermal therapy (reprinted with permission from [184]); (**h**) highly stretchable strain sensors based on double-network hydrogels manufactured using microfluidic devices (reprinted with permission from [185]); (**i**) wearable sensors based on microfluidic conductive hydrogels (reprinted with permission from [186]).

The additive manufacturing (AM) technique involves the layer-by-layer deposition of materials to form three-dimensional structures and has demonstrated significant advantages in fabricating hydrogel-based continuum soft robots [156] (Table 5). Various studies have provided comprehensive insights into AM techniques for biomimetic continuum robotics, enhanced adhesion, hydroelectric actuation, self-healing capability, and conductive hydrogel–elastomer composites [157,158,159,160]. Cheng et al. [161] employed alginate as a rheological modifier in direct ink writing (DIW) to fabricate multifunctional, biocompatible hydrogels, enabling advances in freeform 3D shaping, enhanced mechanical toughness, tunable mechanical properties, and diversified stimulus-responsive behaviors in soft robots (Figure 2a). Kunwar et al. developed high-resolution, stretchable hydrogel structures via digital light processing (DLP) lithography, providing novel pathways toward high-precision manufacturing [162]. Heiden et al. [40] utilized raw materials extracted from industrial waste to synthesize hydrogels and subsequently fabricated hydrogel-based continuum soft robots using 3D printing technology (Figure 2b). Zhang et al. [159] fabricated conductive hydrogels by combining 3D printing and solvent replacement, achieving self-healing capability, ultra-high elongation, and high sensitivity even at temperatures as low as −80 °C (Figure 2c). In comparison, four-dimensional (4D) printing not only retains the strengths of 3D printing but also imparts hydrogels with programmable deformation capabilities under external stimuli such as temperature, pH, humidity, electromagnetic fields, or light [163,164,165]. Liu et al. [166] reported hydrogel scaffolds fabricated via 4D printing exhibiting swelling-induced reinforcement and programmable deformation behaviors, indicating potential applications in minimally invasive implantation (Figure 2d). Solis et al. investigated the effects of printing temperature on the performance of poly(N-isopropyl acrylamide) (pNIPAM) hydrogels produced through 4D DLP printing [167]. Sheikhi et al. [168] leveraged 4D printing to fabricate granular hydrogels possessing self-healing, near-infrared responsiveness, shape-memory behavior, and antibacterial properties, offering novel strategies for control and actuation in hydrogel-based continuum soft robots. Additionally, related studies explored multi-hydrogel 4D printing via direct ink writing within viscous liquids [169], hydrogel actuators designed based on diffusion pathway architecture via 4D printing [170] (Figure 2e), and programmable shape-changing hydrogels for surgical applications [171]. Concurrently, actuator fabrication with soft robotic functionality through 4D printing, humidity-driven hydrogel-based continuum soft robots [172,173], biomimetic applications of multi-material 4D printing [174], and dual-stimuli-responsive intelligent soft carriers were investigated [175] (Figure 2f).

**Table 5:** Fabrication of hydrogel-based continuum soft robots via additives and microfluidics.

Type	Application	Hydrogel	Ref.
3D	Bioelectronics interface	Conductive polymer hydrogel	[176]
	Bioelectronics and tissue engineering	PEDOT:PSS hydrogel	[177]
	Biomimetic soft robotics	DIW printed hydrogel	[161]
	Electroactive soft robotics	Electroactive hydrogel	[157]
	Stretchable electronics	Hydrogel–elastomer composite	[158]
4D	AI soft robotics	AI-based soft module	[178]
	Actuator design	Diffusion-path hydrogel actuator	[170]
	Soft robotic actuator	Functional 4D-printed hydrogel	[172]
	Humidity-responsive robotics	Humidity-driven hydrogel robot	[173]
	Biomimetic shape transformation	Multi-responsive hydrogel	[174]
	Dual stimuli-responsive robotics	Dual stimuli soft carrier	[175]
	Self-healing robotics	Self-healing hydrogel	[179]
Microfluidic	Soft robot actuation	Composite hydrogel fiber	[180]
	Soft robot grasping	Microfluidic micro-gripper	[181]
	Intelligent agent integration	PAAm hydrogel array	[182]
	Integrated control	Light-responsive hydrogel	[183]

### 3.3. Microfluidic Fabrication

Microfluidic technology provides an efficient strategy for fabricating hydrogel-based continuum soft robots by precisely manipulating fluids at the microscale (Table 5). Zhao et al. [187] focused on preparing injectable microfluidic hydrogel microspheres. Ha et al. [184] constructed an electro-responsive hydrogel microfluidic actuator platform based on highly conductive silver nanowires and collagen (Figure 2g). Chai et al. [188] proposed a strategy employing core-shell structured microgels to protect cells during extrusion-based 3D printing of microfluidic microgel scaffolds. Kim et al. [185] utilized microfluidically fabricated double-network hydrogel microfibers, demonstrating exceptional stretchability, high sensitivity, and excellent cyclic stability (Figure 2h). Peng et al. [180] employed microfluidics to fabricate composite hydrogel fibers with porous internal structures and investigated their bending behaviors under a non-contact direct current electric field. Geng et al. [181] developed a temperature-responsive microgel gripper based on microfluidic technology, achieving precise material transport. Sood et al. [189] utilized hydrogel-based microfluidics to construct an effective research platform mimicking the microenvironment of the blood-brain barrier. Jiao et al. [182] integrated polyacrylamide hydrogel arrays within microfluidic devices to enable molecules’ reversible capture and release. Luo et al. [190] developed a novel insulin delivery system employing microfluidics to generate alginate microcapsules encapsulating β-cells. Tolabi et al. [191] discussed the application of microfluidic technologies in cartilage tissue engineering. Zhang et al. [192] prepared hydrogel microspheres for drug delivery using microfluidics. Pan et al. [183] fabricated hydrogel materials capable of rapid volumetric response to photostimulation through microfluidics, enabling integrated control within microfluidic systems. Gao et al. [193], and Chen et al. [194] demonstrated the critical role of microfluidics in the precise fabrication of hydrogel microparticles, particularly those with complex internal architectures. Wang et al. [195] reported synthesizing gelatin hydrogel beads using droplet microfluidics. Xu et al. [186] combined microfluidic design with conductive PEDOT: PSS hydrogels, realizing real-time and highly sensitive detection of uric acid in sweat while simultaneously providing electrolyte storage and enhanced flexibility (Figure 2i).

## 4. Hydrogel-Based Actuators

### 4.1. Solute or Solvent Responsive Actuators

**Figure 3:**
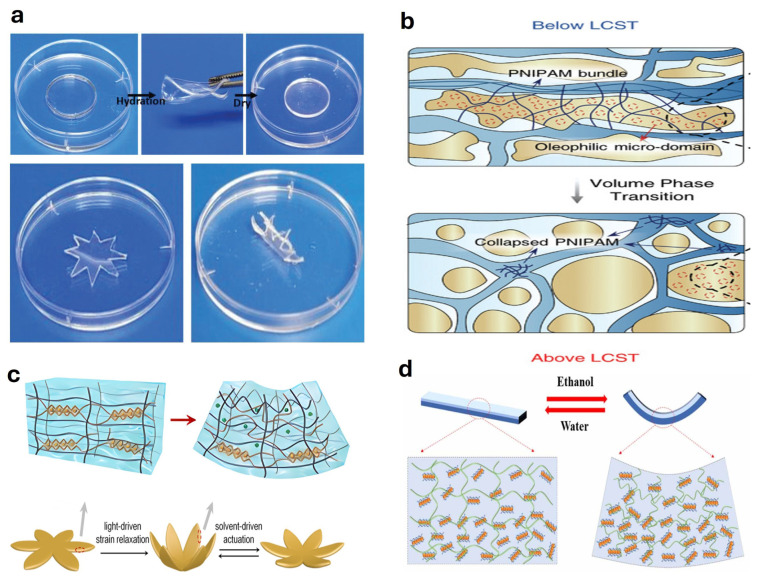
Solute or solvent actuator: (**a**) electrochemical solute actuator (reprinted with permission from [196]); (**b**) physical crosslinking solvent response (reprinted with permission from [198]); (**c**) physical chemical solvent actuator (reprinted with permission from [199]); (**d**) solvent response actuator (reprinted with permission from [202]).

Hydrogel-based solute- or solvent-driven actuators use either the chemical properties of solutes or the physical interactions of solvents to induce material responses, thereby driving mechanical system movements. Ridha et al. [196] approached the issue from a solute perspective and demonstrated that by modulating the concentrations of ions such as $H^{+}$ and ${\mathrm{Fe}}^{3+}$ in the solution, the ionic coordination and hydrogen-bonding crosslinking network can be dynamically tuned, thereby endowing the hydrogel with excellent mechanical properties, self-healing, and shape memory functionalities (Figure 3a). Liang et al. [197] synthesized a fully physically crosslinked hydrogel through radical polymerization followed by immersion. This hydrogel exhibited superior mechanical strength, toughness, and stretchability alongside solvent-responsive capabilities, enabling the design of a soft robotic gripper to grasp objects approximately 1.56 times its weight. Zhang et al. [198] proposed an actuator inspired by skeletal muscles, which achieved high-frequency actuation and remarkable load-bearing capacity by confining solvents within a hydrophobic microenvironment (Figure 3b). Li et al. [199] prepared a sodium alginate/polyacrylamide (SA/PAAm) hydrogel that, upon ultraviolet irradiation, triggered the reduction of ${\mathrm{Fe}}^{3+}$ to ${\mathrm{Fe}}^{2+}$ and the dissociation of the SA network. This significantly decreased the crosslink density and modulus of the hydrogel (Figure 3c). The reaction mechanism-driven network modification and swelling behaviors revealed the complex solvent-driven characteristics of hydrogels. Further research extended this concept to scalable double-network designs [200]. Pinto et al. [201] described a solvent-driven approach focusing on dehydration and shrinkage phenomena observed during the formation of calcium alginate hydrogels. These phenomena were driven by the hydration of biopolymer networks and subsequent solvent release (water). Wang et al. [202] successfully developed a solvent-responsive hydrogel by embedding calcium phosphate oligomer nanoclusters into polyvinyl alcohol (PVA) and sodium alginate matrices. The precise control over the bending angle of this hydrogel was achieved by adjusting the water-to-ethanol ratio, enabling its application as an underwater actuator (Figure 3d). Debta et al. [203] developed functionally gradient hydrogel films that exhibited rapid, reversible shape transformations across different solvent environments by precisely manipulating their internal gradient structures. Parimita et al. [204] reported the 4D printing of two solvent-responsive hydrogels, forming bilayer structures capable of bidirectional actuation depending on solvent pH.

### 4.2. pH Responsive Actuator

**Figure 4:**
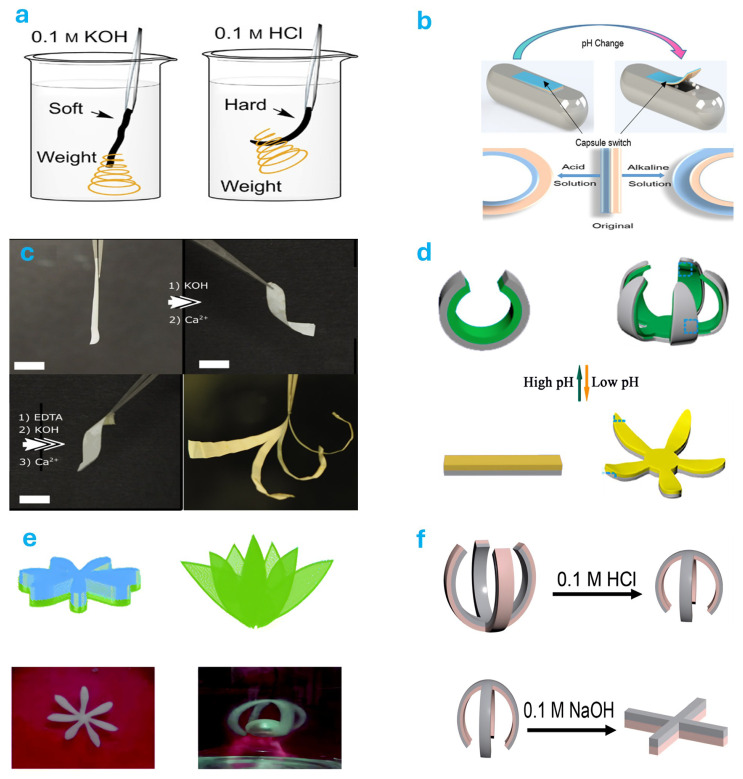
pH-responsive actuators: (**a**) lignin-based pH-responsive hydrogel-based actuators (reprinted with permission from [206]); (**b**) drug delivery pH-responsive hydrogel-based actuators (reprinted with permission from [207]); (**c**) programmable pH-responsive hydrogel-based actuators (reprinted with permission from [208]); (**d**) pH-responsive soft actuators made of heterogeneous hydrogels (reprinted with permission from [210]); (**e**) pH-responsive hybrid double-layer hydrogel-based actuators made of chitosan and PVA (reprinted with permission from [211]); (**f**) pH-responsive hydrogel-based actuators with peptide molecules introduced into PNIPAM (reprinted with permission from [212]).

pH-responsive hydrogel-based actuators achieve actuation by exploiting structural and volumetric changes in hydrogels under varying acidic and basic environments [205]. Dai et al. [206] reported a rapid pH-responsive hydrogel derived from lignin, capable of quickly switching between soft-hard and straight-curved states within one minute, with excellent repeatability (Figure 4a). Han et al. [207] developed a dual pH-responsive hydrogel-based actuator for lipophilic drug delivery (Figure 4b). Houben et al. [208] proposed a reprogrammable, pH-sensitive actuator comprising a polypropylene support with an oriented modulus and a liquid crystal network hydrogel layer that undergoes directional swelling. This actuator exhibits substantial deformation within a narrow pH range (Figure 4c). Yang et al. [209] constructed various heterogeneous hydrogel-based actuators by integrating heterogeneous hydrogels with a pH oscillator. In subsequent studies, by incorporating luminescent agents and pH-responsive hydrogels, they successfully fabricated soft actuators capable of mimicking dynamic jellyfish deformation along with fluorescence color changes [210] (Figure 4d). Additionally, Li et al. [211] described a hybrid bilayer hydrogel-based actuator controlled by pH composed of chitosan and polyvinyl alcohol (PVA). This actuator demonstrated outstanding bending responsiveness and mechanical performance, achieving programmable three-dimensional shape transformations and efficient cargo transport by leveraging asymmetric crystalline region distributions and chemical crosslinking strategies (Figure 4e). Xiang et al. [212] designed a pH-responsive peptide molecule and incorporated it into a poly(N-isopropylacrylamide) (PNIPAM) polymer network, developing a novel hydrogel-based actuator responsive to pH changes. These studies advanced theoretical developments in pH-responsive actuators and provided innovative strategies for practical applications (Figure 4f). Lastly, Lai et al. [213] introduced a pH-responsive hydrogel used to activate a magnetically driven spring-loaded hybrid actuator, which enabled cylindrical hydrogels measuring 3 to 4 mm in diameter to generate nominal blocking forces ranging from 610 to 819 kPa.

### 4.3. Chemical Reaction Actuator

**Figure 5:**
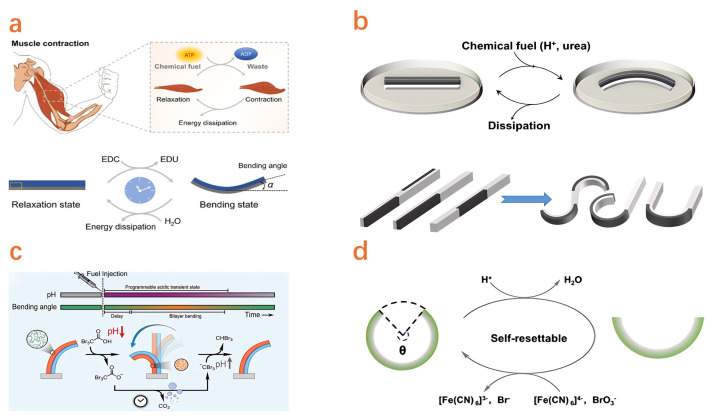
Chemical reaction actuators: (**a**) chemical fuel-driven self-resetting hydrogel-based actuators (reprinted with permission from [215]); (**b**) chemical fuel-controlled double-layer hydrogel-based actuators (reprinted with permission from [216]); (**c**) chemical reaction self-regulating actuators (reprinted with permission from [217]); (**d**) chemical fuel self-resetting double-layer hydrogel-based actuators (reprinted with permission from [218]).

The reaction-driven mechanism of hydrogel-based actuators relies on the reaction between substances embedded within the hydrogel matrix and specific chemical agents, which releases energy to facilitate mechanical output. Wang et al. [214] developed an emulsion hydrogel motor that integrates low-boiling-point oil fuels within a hydrogel matrix, enabling locomotion speeds up to 14.78 mm/s upon thermal stimulation. Furthermore, Xu et al. [215] introduced a chemically fueled, self-resetting hydrogel actuator capable of modulating its operational range and duration by adjusting the amount of fuel, capable of cyclic reuse through replenishment of the chemical fuel (Figure 5a). Zhang et al. [216] described a bilayer hydrogel actuator consisting of a pH-sensitive and non-sensitive layer. This design achieves actuation through stepwise pH regulation, induced by rapid acidification and the urease-catalyzed production of ammonia (Figure 5b). Fusi et al. [217] reported a hydrogel actuator incorporating an integrated pH-feedback system, structurally divided into pH-sensitive and pH-insensitive components. This actuator achieves chemical–mechanical–chemical self-regulation by spontaneously transitioning from one acidic state to another via spontaneous decarboxylation (Figure 5c). Nan et al. [218] developed a novel chemically fueled, self-resetting bilayer hydrogel actuator. Leveraging a negative-feedback reaction network to mimic natural muscle behavior, this actuator exhibits temporary deformation upon adding chemical fuel (Figure 5d). Zhao et al. [219] proposed an ionically fueled soft hydrogel actuator that utilizes coordinated swelling and shrinking behaviors of a Janus bilayer hydrogel, thereby achieving a waste-free, programmable, and reusable actuation mechanism.

### 4.4. Temperature Responsive Actuators

**Figure 6:**
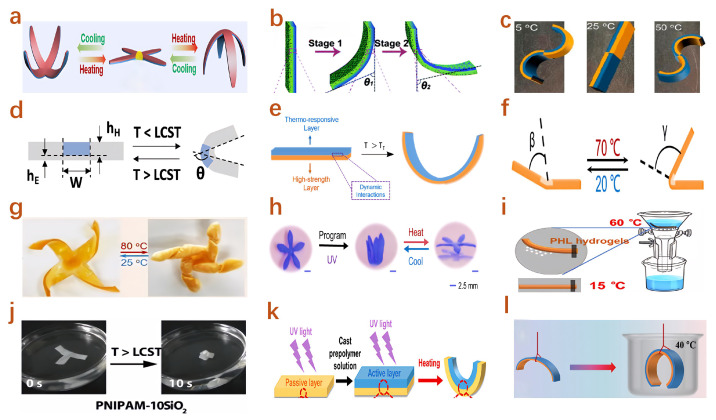
Temperature actuators: (**a**) complementary temperature-responsive double-layer hydrogel-based actuators (reprinted with permission from [220]); (**b**) anisotropic gradient hydrogel temperature-responsive actuators (reprinted with permission from [221]); (**c**) microscale temperature-responsive hydrogel-based actuators (reprinted with permission from [223]); (**d**) NC-PNIPAM hydrogel hinge actuators (reprinted with permission from [224]); (**e**) high-strength hydrogel actuators with biomimetic gradient and ultrafast thermal responsiveness (reprinted with permission from [226]); (**f**) thermally responsive P(NIPAM-co-DMAEMA)/alginate-based hydrogel-based actuators (reprinted with permission from [230]); (**g**) microscale engineering to achieve fine control of the 3D structure of pNIPAM hydrogels (reprinted with permission from [231]); (**h**) photoprogrammed thermoresponsive hydrogel-based actuators (reprinted with permission from [232]); (**i**) temperature-responsive anisotropic hydrogel actuators with inhomogeneous gradient porous structures (reprinted with permission from [233]); (**j**) thermosensitive actuators with asymmetric structures of inorganic particles embedded in PNIPAM hydrogels (reprinted with permission from [234]); (**k**) biodegradable temperature-responsive bilayer actuators (reprinted with permission from [235]); (**l**) PNIPAM-hydrogels with fast responsiveness and excellent mechanical properties (reprinted with permission from [236]).

Temperature-responsive hydrogel-based actuators generate mechanical actuation by swelling or shrinking at specific temperature thresholds. Li et al. [220] developed a bilayer hydrogel-based actuator exhibiting complementary thermal responses, demonstrating antagonistic expansion and contraction behaviors at extreme temperatures (Figure 6a). Liu et al. [221] introduced an anisotropic gradient hydrogel actuator based on poly(N-isopropylacrylamide) (PNIPAM), capable of rapidly responding at 50 °C with adjustable posture changes (Figure 6b). Jiang et al. [222] reported a macroporous thermogel actuator featuring versatile shape reprogrammability. Asoh et al. [223] fabricated microscale temperature-responsive hydrogel actuators through the layered assembly of thermally responsive microgels with varying diameters, enabling bending actuation upon heating and cooling (Figure 6c). Zhao et al. [224] developed a size-tunable NC-PNIPAM hydrogel hinge (Figure 6d). Hong et al. [225] engineered a graphene-containing PEG-PNIPAM hydrogel-based bilayer actuator by modulating the physical properties of PNIPAM-based hydrogels. Li et al. [226] synthesized a high-strength biomimetic gradient hydrogel actuator characterized by ultrafast thermal responsiveness (Figure 6e). Li et al. [227] proposed a thermally responsive soft actuator with spatially programmable Young’s modulus, achieved via grayscale UV light processing, facilitating multiple controllable deformations under global thermal stimuli. Zhang et al. [228] developed a robust and rapidly responsive thermally actuated double-network hydrogel, integrating the thermal sensitivity of PNIPAM and the superior mechanical strength of elastomeric polyurethane (EPU). This hydrogel demonstrated significant deformation within two minutes at 50 °C and was successfully applied in fluid temperature sensing and control. Xiong et al. [229] designed a bilayer wood-based hydrogel actuator combining the structural stability of delignified wood and the temperature-responsive characteristics of AG/PNIPAM composite hydrogels, achieving strong adhesion, swelling resistance, and predictable bending deformation.

Wang et al. [230] developed a thermal-responsive hydrogel actuator based on P(NIPAM-co-DMAEMA)/alginate, characterized by programmable morphology and internal actuating structures (Figure 6f). Spratte et al. [231] created a random network of interconnected microchannels within hydrogels, enabling precise control over the 3D structures of pNIPAM hydrogels, and designed a temperature-driven soft gripper based on this principle (Figure 6g). Zhang et al. [237] reported a structurally graded hydrogel, whose gradient design facilitated precise control over the direction and magnitude of thermally responsive actuation through shape-memory effects after detachment from the substrate. Li et al. [232] introduced a thermal-responsive hydrogel actuator incorporating dynamic photothermal-responsive bonds, capable of rapid heating via near-infrared light, and offering shape programmability as well as remote optical power supply functionality (Figure 6h). Chen et al. [233] fabricated a thermally responsive anisotropic hydrogel actuator featuring a distinctive heterogeneous gradient porous structure by employing a precipitation-driven method (Figure 6i). Fan et al. [234] reported an asymmetric thermosensitive actuator prepared by embedding inorganic particles into PNIPAM hydrogels, wherein the actuating performance could be tailored by varying the type and content of inorganic particles (Figure 6j). Huang et al. [235] developed a biodegradable bilayer hydrogel actuator responsive to temperature stimuli (Figure 6k). Yang et al. [236] reported a PNIPAM-based hydrogel actuator constructed from bilayers with different lower critical solution temperatures (LCSTs), enabling finely tunable hydrogel actuation (Figure 6l).

### 4.5. Light-Responsive Actuators

**Figure 7:**
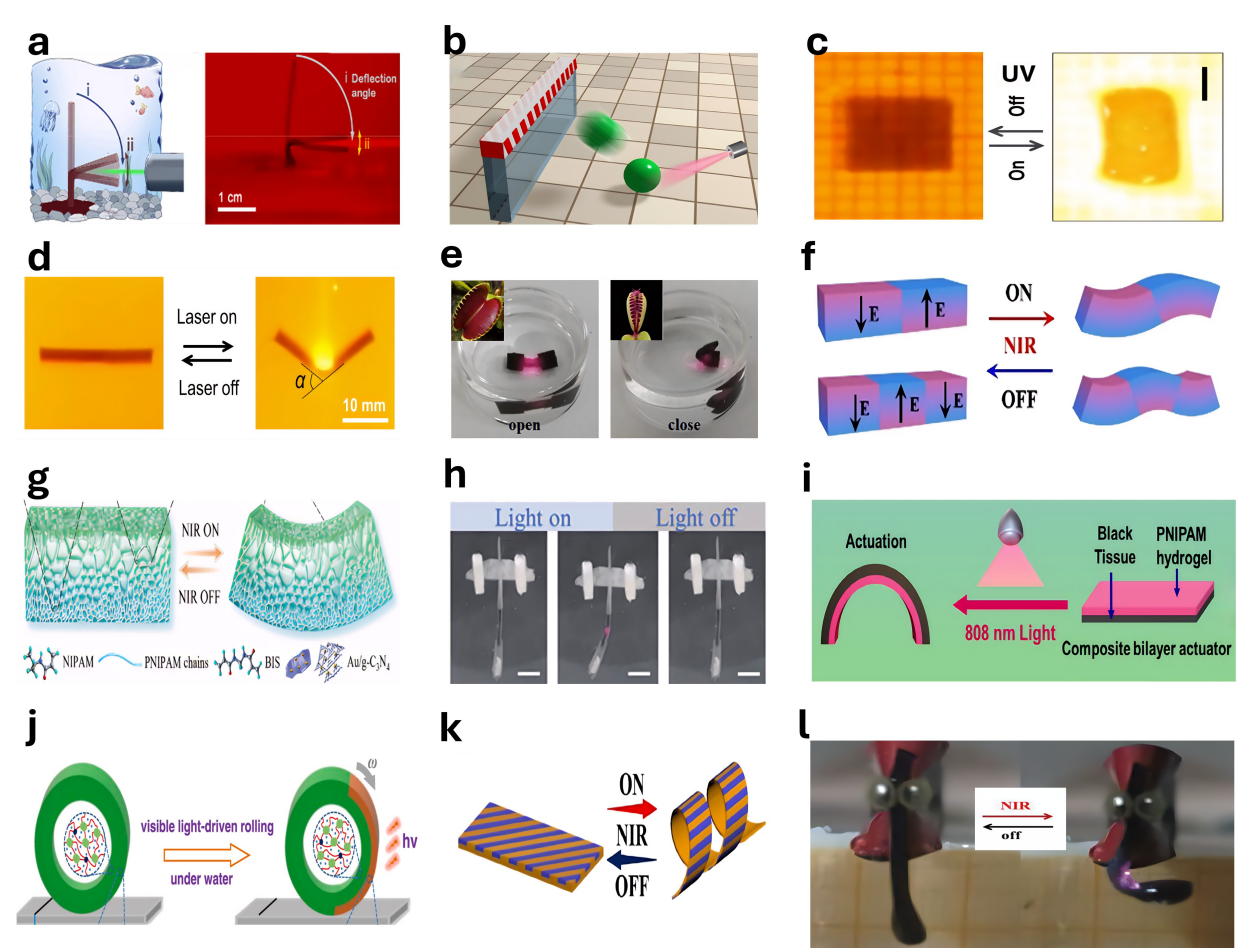
Photohydrogel-based actuators: (**a**) all-soft, photosensitive hydrogel actuators (reprinted with permission from [238]); (**b**) hydrogel-based actuators that can quickly respond to light in air (reprinted with permission from [240]); (**c**) hydrogel-based actuators based on electrical orientation and photolithography polymerization (reprinted with permission from [241]); (**d**) self-driven hydrogel-based actuators under illumination (reprinted with permission from [242]); (**e**) near-infrared responsive hydrogel-based actuators based on PNIPAAm hydrogels [243]; (**f**) near-infrared light-responsive anisotropic hydrogel-based actuators (reprinted with permission from [244]); (**g**) photoresponsive anisotropic hydrogel-based actuator nanoparticles (reprinted with permission from [245]); (**h**) photosensitive flexible actuators composed of PNIPAM/GO hydrogels (reprinted with permission from [247]); (**i**) tissue paper/PNIPAM hydrogel double-layer composite actuator (reprinted with permission from [248]); (**j**) visible-light-driven underwater biomimetic actuator (reprinted with permission from [249]); (**k**) near-infrared (NIR) light-activated hydrogel-based actuator (reprinted with permission from [250]); (**l**) MXene-based near-infrared light irradiation-responsive conductive hydrogel-based actuator (reprinted with permission from [251]).

Photoresponsive hydrogel-based actuators achieve actuation through changes in their physical or chemical structures upon irradiation by specific wavelengths of light. Zhao et al. [238] reported a photosensitive hydrogel actuator used in the fabrication of swimming robots capable of continuous, autonomous phototactic movement under visible light stimulation (Figure 7a). Zheng et al. [239] introduced a micrometer-scale, three-dimensional, light-driven hydrogel-based actuator exhibiting rapid response and outstanding reversibility in water under near-infrared irradiation. Li et al. [240] demonstrated a hydrogel-based actuator capable of fast response to illumination in air, exploiting the photothermal effect of sodium polyacrylate hydrogels embedded with magnetic iron oxide nanoparticles to achieve high-speed jumping and rolling behaviors (Figure 7b). Zhu et al. [241] fabricated patterned hydrogels capable of programmable deformation into three-dimensional structures through a multistep process involving electrical orientation and photolithographic polymerization, achieving complex biomimetic motions such as crawling, walking, and turning (Figure 7c). Further research indicates that various self-driven, continuously moving photodynamic robots can be created by designing asymmetrical structures—such as rotational, mirror-image, spatiotemporal, and chiral symmetry—under illumination (Figure 7d) [242]. Zhang et al. [243] developed a near-infrared-responsive hydrogel-based actuator utilizing PNIPAAm hydrogels, capable of mimicking human gestures, the rapid folding actions of Venus flytraps, and the phototropic behavior of sunflowers (Figure 7e). Xue et al. [244] presented an anisotropic MXene-incorporated hydrogel actuator capable of shape programming, exhibiting diverse geometrical transformations upon near-infrared irradiation (Figure 7f). Chen et al. [245] reported a photoresponsive anisotropic hydrogel-based actuator composed of PNIPAM and gold-coated carbon nitride (Au/g-C3N4) nanoparticles (Figure 7g). Wang et al. [246] developed a highly efficient photo-driven superhydrophobic soft robot employing the photothermal conversion characteristics of TiN@PDMS-LDPE films to achieve rapid, remote-controlled, large-angle deformation under near-infrared laser irradiation, exhibiting self-cleaning properties, corrosion resistance, and amphibious movement capability.

Wang et al. [247] proposed a photosensitive flexible actuator composed of PNIPAM/GO hydrogel. Using the photothermal effect to indirectly trigger the temperature-sensitive properties of the hydrogel, a light-driven micro-swimming fish was developed (Figure 7h). In addition, Wu et al. [248] developed a thin paper/PNIPAM hydrogel double-layer composite actuator. The actuator imitates the light-responsive deformation of organisms. Its surface is also equipped with printed graphite particles, and its tensile strength can reach 1.2 MPa (Figure 7i). Cao et al. [252] reported a hydrogel actuator based on poly (N-isopropylacrylamide-acrylamide)/Fe_3_O_4_ nanocomposite, which exhibited excellent light absorption performance under green light. Xiang et al. [249] developed a visible-light-driven underwater bionic actuator that can achieve curling, twisting, and folding under visible light (Figure 7j). Zhang et al. [250] reported a device that can exhibit a variety of shape change modes under near-infrared light irradiation, including directional bending and chiral twisting. Through periodic near-infrared light irradiation, an octopus-inspired photodynamic soft swimmer was realized (Figure 7k). Yan et al. [253] reported a heterogeneous thermochromic hydrogel film based on photonic nano chains, which is very suitable for artificial muscles and fast-response actuators. Yan et al. [251] prepared an MXene-based conductive hydrogel-based actuator through directional freezing technology. This actuator has an anisotropic structure and can achieve up to 70 % volume shrinkage in just 2 s under near-infrared light irradiation (Figure 7l).

### 4.6. Magnetically Responsive Actuators

**Figure 8:**
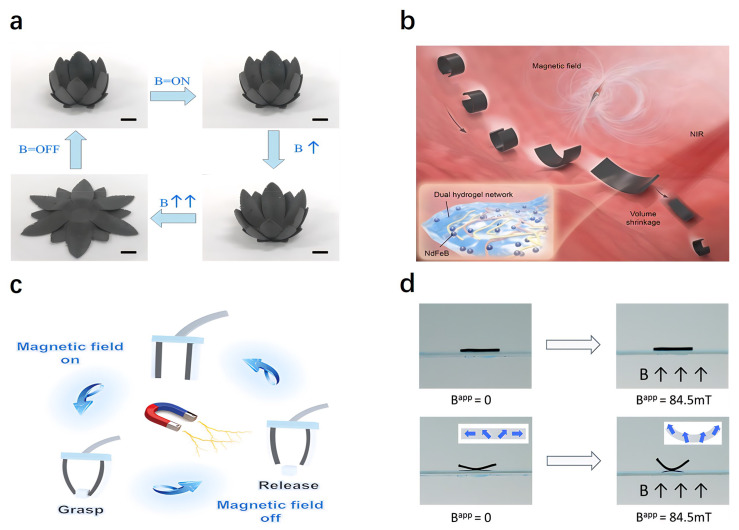
Magnetic actuators: (**a**) 3D printed biomimetic magnetic actuators (reprinted with permission from [254]); (**b**) magnetically driven hydrogel microrobots (reprinted with permission from [255]); (**c**) self-induced magnetically driven bilayer actuators (reprinted with permission from [42]); (**d**) strong magnetic hydrogel-based actuators embedded with hard magnetic particles (reprinted with permission from [256]).

Magnetic responsive hydrogels achieve actuation functions by incorporating magnetically crosslinked particles within their structures, allowing shape and structural control under external magnetic fields. Cao et al. [254] reported a magnetic actuator fabricated using flexible magnetic filament printing technology. They successfully printed biomimetic magnetic actuators capable of emulating natural phenomena, such as octopus tentacle movements, butterfly flight, and plant blooming (Figure 8a). Hou et al. [255] developed a magnetically driven hydrogel micro-robot that exhibits volume-based morphological changes. This robot, fabricated from magnetically patterned hydrogel materials, adopts a “C” shape when placed in a confined liquid environment and achieves forward locomotion through rotational movements induced by an external rotating magnetic field (Figure 8b). Zhang et al. [42] introduced a self-sensing magnetic bilayer actuator composed of magnetic and ionically conductive hydrogels embedded with neodymium-iron-boron magnetic particles. The actuator demonstrates rapid bending deformation responses to magnetic fields, which have been accurately predicted and validated through theoretical analysis and simulation models (Figure 8c). Tang et al. [256] reported a method to enhance the mechanical strength of magnetic hydrogels by embedding hard magnetic particles, making them suitable for high-resolution 3D printing applications (Figure 8d). Chand et al. [257] proposed a mold-free shape-programming technique by incorporating patterned magnetic particles within soft magnetic actuators. This approach enables complex shape programming, allowing actuators to rapidly respond to external magnetic fields with precise deformation and movements.

### 4.7. Electrically Responsive Actuators

**Figure 9:**
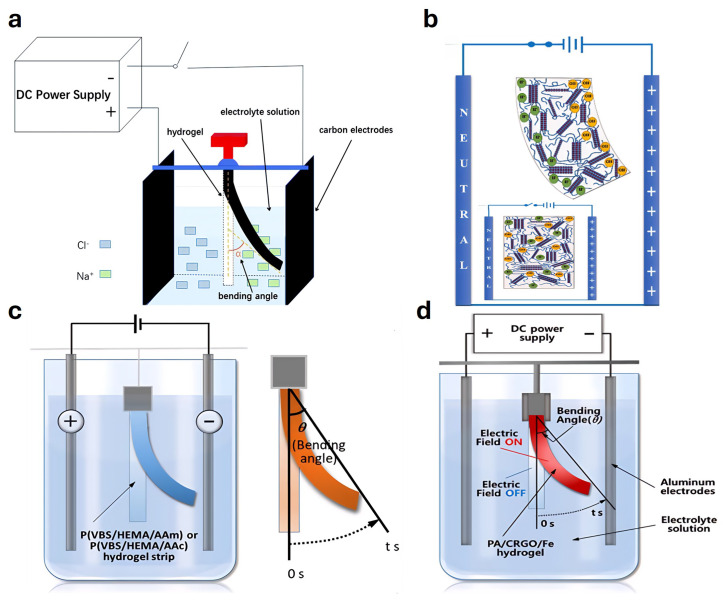
Electro-responsive actuators: (**a**) electro-responsive hydrogel-based actuators constructed with nickel foam (reprinted with permission from [259]); (**b**) physically crosslinked electro-responsive hydrogels (reprinted with permission from [260]); (**c**) low electric field strength hydrogel soft actuators (reprinted with permission from [262]); (**d**) electro-responsive dual network hydrogel-based actuator system (reprinted with permission from [263]).

Electro-responsive hydrogel-based actuators function by exploiting the volume or shape changes of electro-sensitive hydrogels under an applied electric field [258]. Ying et al. [259] developed an electro-responsive hydrogel actuator characterized by employing nickel foam as the structural framework. A conductive network incorporating carbon nanotubes and a physically interlocking network were constructed within this actuator, demonstrating stable and reversible bidirectional bending capability (Figure 9a). Additionally, Ha et al. [184] proposed a microfluidic actuator platform utilizing electro-responsive hydrogels, effectively achieving actuation functions by applying electrical signals to embedded conductive hydrogels (Figure 9g). Rotjanasuworapong et al. [260] synthesized AG hydrogels via a simple solvent-casting technique and demonstrated their outstanding electroactive response capability, indicating their suitability for hydrogel-based actuators (Figure 9b). Hong et al. [261] introduced a flexible actuator based on conductive polyacrylamide hydrogels, utilizing their high water retention, exceptional stretchability, and excellent electrical conductivity to fabricate flexible electrode materials. Shin et al. [262] developed an electrically responsive soft actuator fabricated from monomers VBS, HEMA, AAm, and AAc, achieving dynamic and precise control under low electric field intensities, thus being suitable for various human–machine interface applications (Figure 9c). Lee et al. [263] designed an electro-responsive double-network hydrogel-based actuator, exhibiting superior stretchability, mechanical performance, self-healing capability, and rapid electrical responsiveness, with biocompatibility validated through in vitro cytotoxicity assays (Figure 9d).

### 4.8. Hydraulic or Electro-Osmotic Actuators

**Figure 10:**
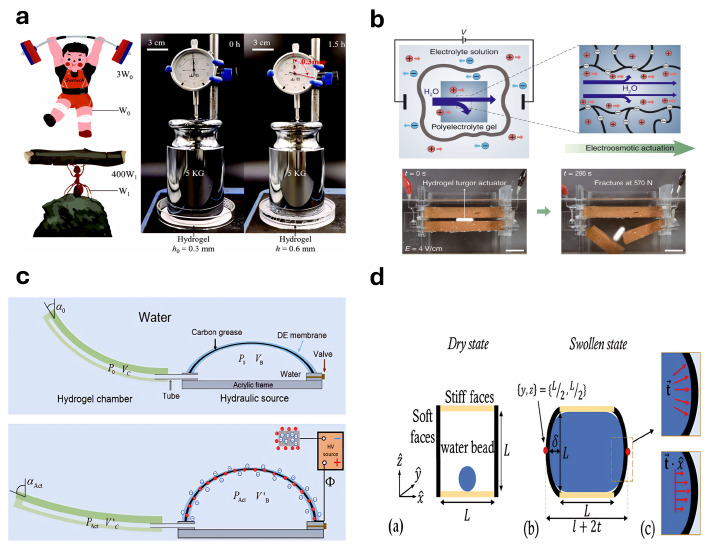
Hydraulic or electro-osmotic pressure actuators: (**a**) osmotic swelling stress actuators (reprinted with permission from [265]); (**b**) hydrogel-based actuators with swelling pressure and electro-osmotic effects (reprinted with permission from [266]); (**c**) soft hydraulic robotic actuators (reprinted with permission from [267]); (**d**) devices for measuring swelling stress generated when a gel absorbs solvent under mechanical constraints (reprinted with permission from [268]).

Hydraulic actuation relies on the transfer of energy via changes in pressure within hydrogel-based fluids. In contrast, electro-osmotic actuation induces fluid movement through the interaction between charged particles or fluids and electric fields in hydrogel matrices. Although these two mechanisms differ significantly in principles and applications, both operate by controlling energy input to generate pressure gradients that drive the motion of working fluids. Yuk et al. [264] developed a hydrogel-based actuator driven hydraulically by multiple programmable high-throughput injection pumps that supply pressurized water. Compared to osmosis-driven hydrogel actuators, this hydraulic actuator achieves incredible speed and force and enables optical and acoustic camouflage in underwater environments. He et al. [265] significantly enhanced the osmotic swelling stress by optimizing the composition and structure of polyacrylamide hydrogels, elevating the actuator stress from approximately 180 kPa to around 400 kPa. They demonstrated a hydrogel jack capable of lifting weights to 2000 times its mass using the amplified osmotic swelling stress (Figure 10a). Na et al. [266] reported a hydrogel actuator that integrates osmotic swelling pressure with electro-osmotic effects, effectively converting high osmotic pressure into a substantial actuator force. Additionally, active water transport into the hydrogel via electro-osmotic effects significantly increased the actuator’s speed and strength, enabling it to fracture rigid bricks within minutes (Figure 10b). Zhang et al. [267] designed a soft hydraulic robotic actuator that combines dielectric elastomers with hydrogels. In this design, an inflatable dielectric elastomer balloon is a soft hydraulic source capable of adjusting the internal water pressure through applied voltage. At the same time, connected hydrogel chambers function as hydraulic actuators, facilitating the deformation of the soft robot’s gripping components (Figure 10c). Levin et al. [268] described the design of an experimental apparatus for measuring swelling-induced stress generated when gels absorb solvents under mechanical constraints (Figure 10d). Hu et al. [269] developed a twisted and coiled hydrogel fiber muscle actuated by electrolyte-driven osmotic flow. Incorporating semipermeable polymer tubes to enhance water transport efficiency improved contraction stroke rates and driving stresses, enabling faster and stronger actuation performance.

### 4.9. Humidity Responsive Actuators

**Figure 11:**
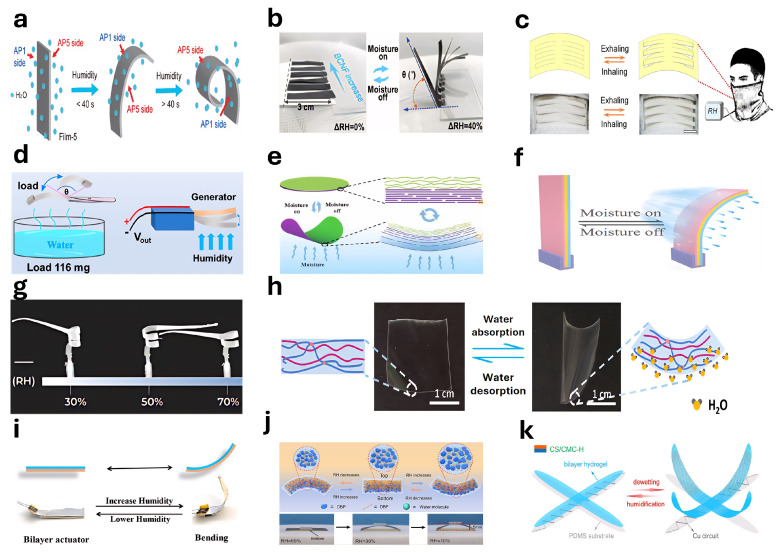
Humidity actuator: (**a**) a humidity-sensitive soft actuator based on the ratio of sodium alginate (SA) and polyvinyl alcohol (PVA) (reprinted with permission from [274]); (**b**) a humidity actuator with high conductivity and humidity response capability (reprinted with permission from [275]); (**c**) a double-layer HAMA/PVDF membrane humidity-sensitive actuator (reprinted with permission from [276]); (**d**) a humidity-driven photochromic self-oscillating actuator based on SP@AG material (reprinted with permission from [278]); (**e**) a humidity-responsive actuator based on MXene (reprinted with permission from [279]); (**f**) a three-layer structure humidity actuator using the yin-yang interface (reprinted with permission from [280]); (**g**) a 4D printed humidity-sensitive actuator (reprinted with permission from [173]); (**h**) a visual and programmable humidity-driven thin film actuator (reprinted with permission from [281]); (**i**) self-supported humidity-responsive actuators prepared by using rigid covalent organic frameworks and flexible polyethylene glycol polymers (reprinted with permission from [282]); (**j**) biocompatible and biodegradable DB film humidity-responsive actuators (reprinted with permission from [283]); (**k**) two-in-one flexible devices integrating humidity actuator and humidity-responsive sensor functions (reprinted with permission from [284]).

Hydrogel-based humidity actuators are devices capable of sensing changes in environmental humidity and generating mechanical motion via alterations in shape or structure [270,271,272,273]. Tan et al. [274] successfully fabricated a humidity-sensitive actuator by adjusting the ratio of sodium alginate (SA) to polyvinyl alcohol (PVA) and assembling these materials into a multilayer laminated structure (Figure 11a). Yang et al. [275] reported an efficient actuator capable of achieving rapid, reversible, and controllable large-scale deformation under low-humidity gradients (Figure 11b). Tang et al. [276] developed a bilayer HAMA/PVDF membrane humidity-sensitive actuator used as an innovative ventilation valve designed to regulate relative humidity in outdoor masks effectively (Figure 11c). Additionally, Tang et al. [277] presented a hydrogel-based actuator for food preservation realized by integrating glycerol-crosslinked polyvinyl alcohol with porous polyvinylidene fluoride. Wang et al. [278] reported a humidity-driven actuator based on SP@AG materials capable of sustained oscillations driven by humidity-induced photochromism (Figure 11d). Hou et al. [279] introduced a novel MXene-based humidity-responsive actuator utilizing a heterogeneous structure composed of stacked MXene nanosheets and 3D crumpled MXene films (Figure 11e). Li et al. [280] reported a novel tri-layer actuator with a core design featuring an ionic interface, enabling substantial oscillatory movements even under slight temperature variations (Figure 11f). Cecchini et al. [173] employed 4D printing technology and hygroscopic polymer materials to create a humidity-responsive actuator mimicking seed locomotion behaviors, capable of autonomous movement driven by ambient humidity changes (Figure 11g). Guo et al. [281] fabricated a visualizable and programmable humidity-driven thin-film actuator using polyvinyl alcohol (PVA), polyacrylamide (PAAm), and tannic acid (TA)(Figure 11h). Mao et al. [282] synthesized a humidity-responsive actuator by integrating rigid covalent organic frameworks (COFs) with flexible polyethylene glycol (PEG) polymer chains, capable of generating electrical energy through continuous self-oscillatory movements (Figure 11i). Chen et al. [283] described a biodegradable humidity-responsive actuator film featuring a gradient structure, whose actuating performance can be adjusted by modulating film thickness (Figure 11j). Zeng et al. [284] reported a robot integrating both humidity actuator and humidity-responsive sensor functions in one device (Figure 11k). Cheng et al. [285] developed a highly efficient humidity-driven bilayer hydrophilic porous composite nanofiber membrane actuator, significantly enhancing humidity-responsive performance through optimized nanofiber orientation and integrated breathing-type MOF, enabling programmable actuation.

### 4.10. Multifunctional Actuator

**Figure 12:**
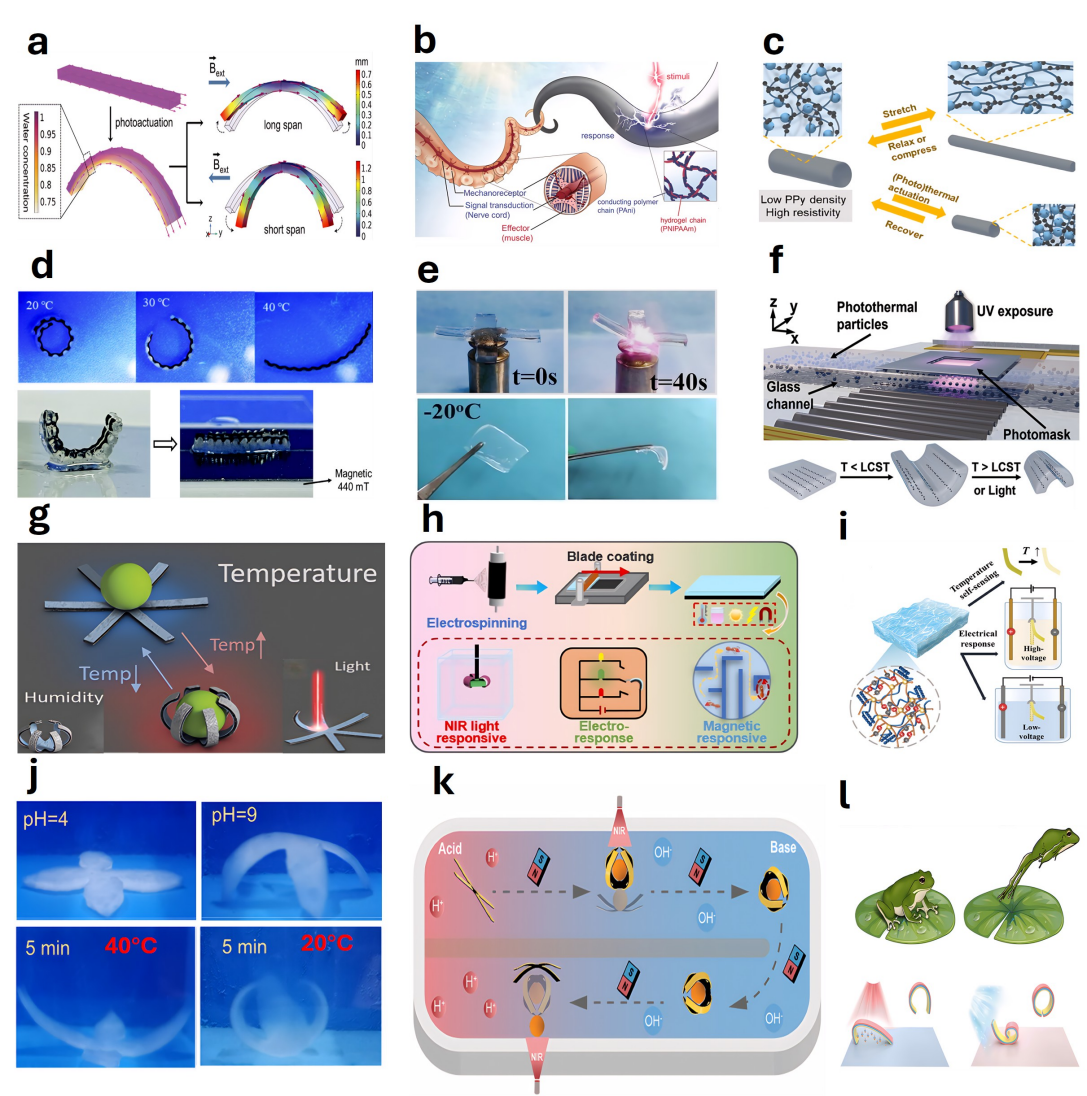
Multi-responsive actuators: (**a**) hydrogel-based actuators that can be rapidly programmed under light and magnetic field stimulation (reprinted with permission from [286]); (**b**) hydrogel-based actuators with electrical conductivity and photothermal responsiveness (reprinted with permission from [288]); (**c**) self-sensing actuators that integrate driving and sensing (reprinted with permission from [289]); (**d**) a multi-responsive double-layer hydrogel-based actuator based on PNIPAM-PEGDA was developed (reprinted with permission from [290]); (**e**) photothermal responsive hydrogels that integrate actuators and sensors (reprinted with permission from [291]); (**f**) composite hydrogel-based actuators developed by acoustic lithography (reprinted with permission from [293]); (**g**) multi-responsive actuators developed by laser scribing technology and electrodeposition method (reprinted with permission from [294]); (**h**) anisotropic bilayer hydrogel-based actuators (reprinted with permission from [295]); (**i**) Agar-Zwitterion hydrogel-based actuators (reprinted with permission from [43]); (**j**) temperature and pH responsive heterogeneous hydrogel-based actuators (reprinted with permission from [296]); (**k**) 4D-printed pH and temperature responsive integrated motion control, actuators and sensors (reprinted with permission from [297]); (**l**) light–humidity dual-responsive synergistic actuators (reprinted with permission from [298]).

Multifunctional hydrogel actuators are continuous hydrogel-based soft robotic devices capable of responding to multiple external stimuli or a single stimulus to achieve actuation and other functionalities such as sensing. Li et al. [286] demonstrated a hydrogel-based actuator capable of rapid programming in response to both light and magnetic fields by embedding ferromagnetic nanowires into photoresponsive hydrogels (Figure 12a). Zhang et al. [287] developed solvent- and thermo-responsive PNIPAAm-based hydrogel actuators. Zhao et al. [288] fabricated a hydrogel actuator with conductive and photothermal responsiveness capable of not only performing actions such as contraction, bending, shape morphing, gripping, and transport but also self-monitoring functionalities (Figure 12b). Lo et al. [289] reported a self-sensing actuator by synthesizing conductive PPy networks in situ within PNIPAAm nanohydrogels, integrating actuation and sensing into a single self-monitoring device (Figure 12c). Wang et al. [290] developed a multi-responsive bilayer hydrogel actuator based on PNIPAM-PEGDA, capable of sensitive responses to temperature, solvent composition, and magnetic fields (Figure 12d). Sun et al. [291] reported a photothermal-responsive hydrogel-based actuator and sensor fabricated successfully via a one-pot polymerization approach combined with calcium chloride (Figure 12e). Wu et al. [292] developed an electrothermal and magnetically actuated shape-memory micro-gripper, integrating electrothermally triggered shape-memory effects with magnetic responsiveness to achieve sequential gripping and release without the need for continuous magnetic fields.

Saadli et al. [299] developed a multi-responsive micro-actuator based on PNIPAm hydrogel, in which embedding oriented magnetite nanoparticles achieved tunable shape transformations and magnetic responsiveness. Zheng et al. [294] designed a multi-responsive actuator capable of sensing and responding to six environmental stimuli, including humidity, temperature, light, radiofrequency heating, low-frequency magnetic fields, and chemical solvents, enabling soft micro-robotic functionalities (Figure 12g). Wei et al. [295] fabricated an anisotropic bilayer hydrogel-based actuator characterized not only by exceptional mechanical strength but also by its capacity to exhibit complex deformations in response to optical, electrical, and magnetic stimuli (Figure 12h). Yang et al. [43] developed an Agar-Zwitterionic hydrogel-based actuator with intrinsic temperature-sensing capability and responsiveness to electrical stimulation (Figure 12i). Jiang et al. [296] reported a temperature- and pH-responsive heterogeneous hydrogel actuator fabricated via a bilayer strategy (Figure 12j). Zhou et al. [297] introduced a 4D-printed hydrogel integrating actuator and sensor functionalities, responsive to variations in pH and temperature (Figure 12k). Li et al. [298] described a synergistic actuator responsive to both light and humidity, wherein the photothermal expansion layer expands upon illumination, while the moisture-responsive layer contracts due to evaporation (Figure 12l). Kong et al. [300] developed a Drosera-inspired bilayer hydrogel-based actuator incorporating dual responsiveness to temperature and humidity.

### 4.11. Perspectives

Hydrogel-based actuators have demonstrated significant potential in diverse fields, including robotics and smart devices. Besides the previously mentioned stimuli-responsive hydrogel actuators, emerging actuator types, such as touch-responsive actuators [301], have also attracted increasing research attention. Currently, the advancement of hydrogel actuators follows three primary directions. First, biodegradable and environmentally friendly hydrogel-based actuators have emerged, typically fabricated from natural polymers or biodegradable synthetic polymers. These actuators reduce environmental impacts and align with global trends toward sustainability. Second, multi-responsive hydrogel actuators are developing to meet the demands of increasingly complex and dynamic application environments. Such actuators can respond to multiple external stimuli, enabling more flexible and precise control. Finally, research has expanded toward integrated innovative hydrogel systems based on multi-responsive actuators. These integrated systems combine sensing and motion-control functionalities, achieving sophisticated self-driven and self-regulated capabilities. This evolution represents a paradigm shift from single-function actuators toward multifunctional intelligent systems.

## 5. Hydrogel-Based Sensors

### 5.1. Strain Sensors

**Figure 13:**
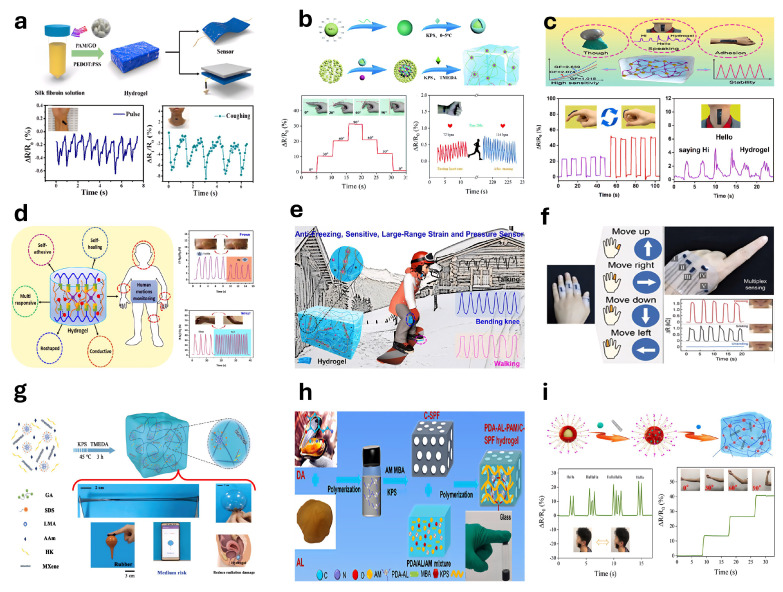
Strain sensors: (**a**) wearable strain sensors [302]; (**b**) highly sensitive strain sensors based on SiO_2_@PANI (reprinted with permission from [303]); (**c**) a novel strain sensor made by combining sodium caseinate (SC) and graphene oxide (rGO) in polyacrylamide (PAAm) hydrogel (reprinted with permission from [305]); (**d**) flexible strain sensors prepared by combining sodium alginate (SA) and polyvinyl alcohol (PVA) (reprinted with permission from [306]); (**e**) strain sensors based on highly elastic and tough chitosan-poly(hydroxyethyl acrylate) double network hydrogel (reprinted with permission from [307]); (**f**) strain sensors based on PEDOT:PSS nanofibers and PVA (reprinted with permission from [311]); (**g**) strain sensors for monitoring radiation damage to organs around tumors caused by radiotherapy (reprinted with permission from [312]); (**h**) lignin-based pressure sensor (reprinted with permission from [313]); (**i**) dual network structure strain sensor (reprinted with permission from [314]).

Hydrogel-based strain/pressure sensors detect external mechanical changes using hydrogel materials and convert these variations into electrical signals for monitoring. He et al. [302] developed a conductive hydrogel for wearable strain/pressure sensors by integrating polyacrylamide and silk fibroin into an elastic matrix and incorporating a mixture of graphene oxide and PEDOT:PSS (Figure 13a). Li et al. [303] reported a highly sensitive strain sensor fabricated by uniformly dispersing silica@polyaniline (SiO_2_@PANI) core-shell particles within a hydrogel, suitable for monitoring human motion (Figure 13b). Sun et al. [304] developed a sensitive MXene nanosheet-based strain sensor and designed an anisotropic bilayer hydrogel actuator capable of rapid and controllable photo-driven bending. Wang et al. [305] described a strain sensor fabricated by incorporating sodium caseinate and graphene oxide into a polyacrylamide (PAAm) hydrogel, demonstrating precise detection capabilities for human vocalization and limb movements; see Figure 13c. Zhao et al. [306] introduced a multifunctional composite hydrogel consisting of sodium alginate (SA) and polyvinyl alcohol (PVA), which effectively serves as a flexible strain sensor for monitoring wrist movements and facial expressions such as frowning; see Figure 13d. Yang et al. [307] utilized ionic solution treatments to prepare double-network hydrogels capable of extensive strain and pressure sensing, accurately capturing subtle human motions and physical states even at subzero temperatures (Figure 13e). He et al. [308] synthesized an organic hydrogel for strain sensors via a binary water/glycerol solvent method under ultraviolet irradiation. Zhang et al. [309] exploited the crystalline properties of PVA to fabricate ultrathin, flexible hydrogel films intended for strain sensing applications. Wang et al. [310] developed a silk fibroin-based biocomposite hydrogel strain sensor, establishing a stable conductive network by incorporating tannic acid and MXene nanosheets.

Shen et al. [311] reported a strain sensor fabricated through a one-step synthesis of PEDOT:PSS nanofibers and polyvinyl alcohol (PVA). The sensor was produced by integrating conductive polymer hydrogels with 3D printing and freeze–thaw techniques, demonstrating high elasticity and negligible hysteresis (Figure 13f). Guan et al. [312] described a conductive hydrogel strain sensor fabricated via a one-step copolymerization method capable of simulating radiation signal attenuation (Figure 13g). Chen et al. [313] reported an ultra-sensitive pressure sensor made from a viscous hydrogel containing polydopamine (PDA), obtained through the slow oxidative polymerization of dopamine (DA) facilitated by aminated lignin (AL). This sensor employed C-SPF carbon aerogel as a substrate combined with a dual-network composite hydrogel composed of PDA, AL, and polyacrylamide (PAM) (Figure 13h). Xie et al. [314] introduced a hydrogel strain sensor fabricated using carboxylated multi-walled carbon nanotubes, resulting in a dual-network structure characterized by hydrogen bonding crosslinks and hydrophobic interactions (Figure 13i).

### 5.2. Pressure Sensors

**Figure 14:**
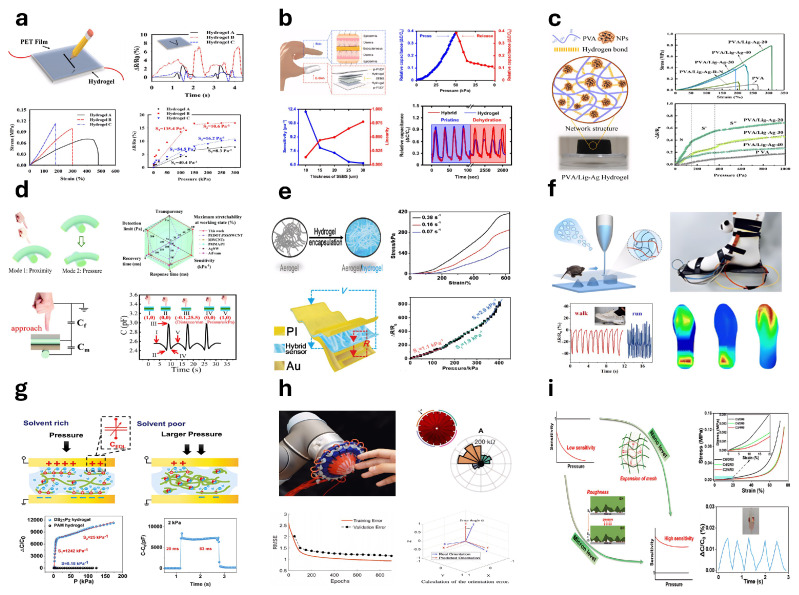
Pressure sensor: (**a**) nano-regulated hydrogel pressure sensor (reprinted with permission from [315]); (**b**) skin-mimicking pressure sensor (reprinted with permission from [316]); (**c**) piezoresistive pressure sensor based on a nanocomposite hydrogel (reprinted with permission from [317]); (**d**) hydrogel pressure sensor for accurate monitoring of plantar pressure (reprinted with permission from [319]); (**e**) a hybrid pressure sensor of aerogel and hydrogel (reprinted with permission from [320]); (**f**) eight-channel pressure sensor for monitoring gait (reprinted with permission from [321]); (**g**) dielectric layer used as ionic pressure sensor (reprinted with permission from [322]); (**h**) multi-axis force sensor for robot end (reprinted with permission from [324]); (**i**) cellulose ion-conductive hydrogel for piezoresistive pressure sensor (reprinted with permission from [323]).

Hydrogel-based pressure sensors consist of flexible hydrogels—soft, porous polymer networks with high water content—that convert mechanical pressure into measurable signals, enabling applications in various sensing technologies. Ryplida et al. [315] developed hydrogels with tunable internal hydrophilic and hydrophobic structures by adjusting nanoparticle hydrophobicity for use in pressure sensors (Figure 14a). Kang et al. [316] fabricated a skin-mimicking sensor using polyacrylamide/sodium alginate hydrogels combined with p-PVDF-HFP-DBP polymer (Figure 14b). Han et al. [317] developed a piezoresistive nanocomposite hydrogel pressure sensor using organic–inorganic hybrid nanoparticles synthesized from alkali lignin and silver nanoparticles, incorporated into a polyvinyl alcohol matrix (Figure 14c). Liu et al. [318] introduced a flexible hydrogel sensor array for precise plantar pressure monitoring and gait pattern identification, enhancing rehabilitation. Ding et al. [319] developed hydrogel fiber-based sensors with polyacrylamide-alginate double-network hydrogels (Figure 14d). Huang et al. [320] described a hybrid pressure sensor designed for fatigue detection (Figure 14e). Zheng et al. [321] employed 3D printing to create microstructured, flexible MRDN hydrogel sensors integrated into wearable insoles for gait analysis (Figure 14f). Zhao et al. [322] synthesized stable ionic hydrogels for dielectric layers in ionic pressure sensors (Figure 14g). Chen et al. [323] presented a multi-axis force sensor using gelatin-based ionic hydrogels and 3D printing combined with recurrent neural networks (Figure 14h). Cai et al. [324] developed optimized cellulose ionic conductive hydrogels (ICH) via a zero-waste approach for ultra-sensitive pressure sensing (Figure 14i). Wang et al. [325] reported a capacitive pressure sensor utilizing solvent-exchanged porous lignin–cellulose hydrogels (SPLCH).

### 5.3. Humidity-Sensitive Hydrogel-Based Sensors

**Figure 15:**
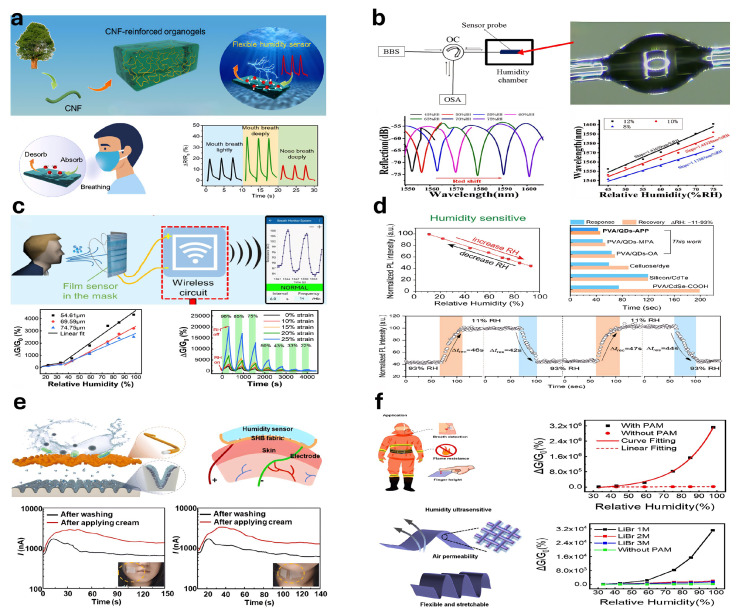
Humidity sensor: (**a**) CS-NC organogel was constructed for a humidity sensor (reprinted with permission from [327]); (**b**) Fabry–Perot (FP) relative humidity (RH) sensor based on optical fiber (reprinted with permission from [328]); (**c**) stretchable transparent humidity sensor based on PAM hydrogel (reprinted with permission from [330]); (**d**) PL-type humidity sensor based on PVA/quantum dot (QD) composite gel (reprinted with permission from [332]); (**e**) ultrathin layered hydrogel–carbon nanocomposite used as wearable humidity sensor (reprinted with permission from [333]); (**f**) in situ synthesis of polyacrylamide (PAM) hydrogel on cotton fabric with flame retardancy, stretchability, breathability, and high-sensitivity humidity sensing fabric (reprinted with permission from [335]).

Hydrogel-based humidity sensors convert environmental humidity variations into electrical signals by exploiting the humidity-sensitive properties of hydrogels [326]. Yu et al. [327] fabricated a CS-NC organogel humidity sensor using a one-pot solvent-exchange method, integrating sorbitol and CaCl_2_ into the hydrogel network (Figure 15a). Xia et al. [328] developed a fiber-optic Fabry–Perot (FP) humidity sensor based on PNIPAM hydrogel, with sensitivity depending on the PNIPAM concentration (Figure 15b). Wu et al. [329] produced humidity sensors from hydrogel films of various thicknesses via spin-coating. Liang et al. [330] constructed a stretchable, transparent humidity sensor from PAM hydrogels enhanced by structural optimization and tapioca starch crosslinking (Figure 15c). Zhang et al. [331] introduced a capacitive humidity sensor based on PAAm hydrogels, improved by glycerol addition, achieving high sensitivity within 12–95% RH. Cheng et al. [332] developed a photoluminescent (PL) humidity sensor from a PVA/quantum dot composite gel, demonstrating excellent sensitivity, rapid response, and mechanical toughness through a droplet-assisted fabrication method (Figure 15d). Pan et al. [333] fabricated an ultrathin hydrogel–carbon composite humidity sensor using solvent-free CVD, suitable for real-time respiration and skin humidity monitoring due to its sensitivity, rapid response, and durability (Figure 15e).

Cesnik et al. [334] employed two-photon polymerization (2PP) 3D printing and initiated chemical vapor deposition (iCVD) to produce ultrathin humidity sensors. Yang et al. [335] synthesized a flame-retardant, stretchable, breathable humidity sensor using PAM hydrogels on cotton fabric, exhibiting an over 311-fold conductivity increase from 11 % to 98 % RH (Figure 15f). Ding et al. [336] developed a PVA-CNF hydrogel humidity sensor via a one-pot spin-coating method, with enhanced performance at reduced film thickness, offering faster response and recovery. Liu et al. [337] created a humidity-adaptive hydrogel integrating PVA, BH, LiCl, and lysozyme (LE). Han et al. [338] designed a humidity sensor from porous hydrogels, graphene oxide, and citral for food preservation. Song et al. [339] introduced an ionic conductive hydrogel incorporating hydroxypropyl cellulose (HPC), enhancing humidity detection, flexibility, and biocompatibility, enabling the precise monitoring of environmental humidity and human respiration.

### 5.4. Conductive Hydrogel-Based Sensors

**Figure 16:**
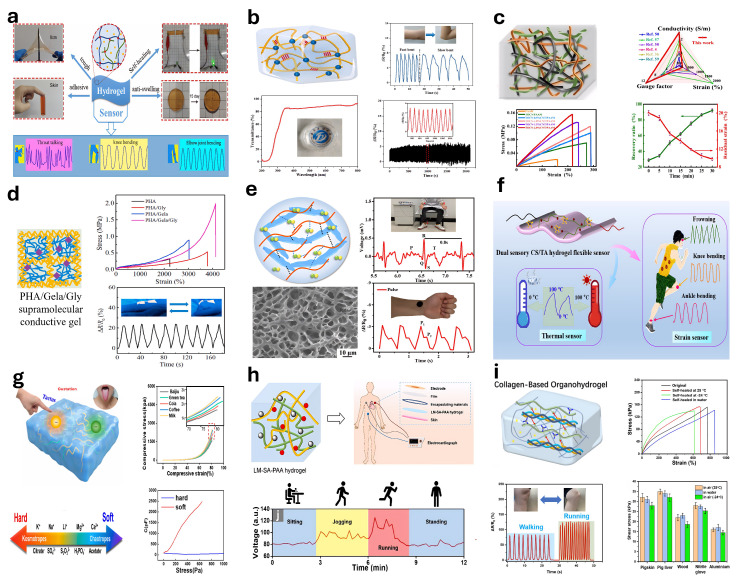
Conductive hydrogel sensor: (**a**) PAA/Fe system was added with 2-hydroxypropyltrimethylammonium chloride chitosan (HACC) to construct a 3+ ion crosslinked network conductive hydrogel sensor (reprinted with permission from [341]); (**b**) two-step synthesis of PAMPS conductive hydrogel for real-time strain sensor (reprinted with permission from [343]); (**c**) in situ free radical polymerization to prepare TOCN-CNT/PAAM nanocomposite conductive hydrogel for electronic skin sensor (reprinted with permission from [344]); (**d**) PHA/Gela/Gly supramolecular conductive hydrogel with double network structure for strain sensor (reprinted with permission from [345]); (**e**) conductive MXene nanocomposite hydrogel for biosensor (reprinted with permission from [346]); (**f**) conductive hydrogels that are highly sensitive to both temperature and strain (reprinted with permission from [347]); (**g**) gel ion–electronic sensor interface based on Hofmeister effect, suitable for taste and touch sensors (reprinted with permission from [348]); (**h**) multifunctional hydrogel (LM-SA-PAA) biosensor (reprinted with permission from [350]); (**i**) collagen-based conductive organic hydrogel CDPAP, used for strain sensors (reprinted with permission from [351]).

Conductive hydrogel sensors are porous polymer networks incorporating conductive fillers, integrating flexibility, biocompatibility, self-healing capabilities, and excellent electrical performance, thus providing high sensitivity and superior mechanical properties in stress–strain sensing applications. An et al. [340] described a dual-network ionic conductive hydrogel temperature sensor. Wang et al. [341] synthesized a conductive multifunctional hydrogel via one-step polymerization for wearable sensing applications, constructing a trivalent ion-crosslinked network by adding 2-hydroxypropyl trimethyl ammonium chloride chitosan (HACC) to a PAA/Fe system (Figure 16a). Ding et al. [342] developed a PAM/Carboxymethyl Chitosan (CMC)/NaCl ionic conductive hydrogel strain sensor through a one-step method. Mao et al. [343] fabricated an adhesive, transparent, stretchable, and strain-sensitive conductive hydrogel suitable for real-time human motion monitoring sensors. This hydrogel employs a two-step synthesized PAMPS, forming a dual-network structure stabilized by covalent crosslinks and hydrogen bonding (Figure 16b). Lu et al. [344] reported an in situ radical polymerization of TOCN-CNT/PAAM nanocomposite conductive hydrogels, applicable for electronic skin sensors (Figure 16c). Yang et al. [345] synthesized a supramolecular conductive hydrogel (PHA/Gela/Gly) featuring a dual-network structure via a one-pot approach suitable for strain sensing and motion detection. The hydrogel combines self-crosslinked PHA chains with gelatin interactions, forming a rigid, freeze-resistant physical network (Figure 16d). Li et al. [346] developed a highly stretchable, self-healing, biodegradable, and biocompatible conductive MXene nanocomposite hydrogel for sensitive human-motion detection and wireless electrophysiological signal monitoring (Figure 16e). Kang et al. [347] reported a conductive hydrogel highly sensitive to temperature and strain, prepared by infiltrating tannic acid (TA) into a crosslinked chitosan (CS) network in an acidic aqueous solution (Figure 16f). Li et al. [348] introduced a gel ion–electronic sensor interface based on the Hofmeister effect, suitable for tactile and gustatory sensors (Figure 16g). Liu et al. [349] proposed a biomass-based conductive hydrogel electronic skin developed using a top–down strategy for wearable sensors. Zhou et al. [350] fabricated a multifunctional conductive hydrogel sensor (LM-SA-PAA) composed of liquid metal (LM), sodium alginate (SA), and polyacrylic acid (PAA) for human motion and ECG monitoring applications (Figure 16h). Ling et al. [351] developed a collagen-based conductive organic hydrogel (CDPAP) for strain sensors and electronic skin (Figure 16i). Zhang et al. [352] prepared an ionic conductive hydrogel using a water/glycerol-based adhesive solvent for strain sensors. Lee et al. [353] synthesized a conductive hydrogel for electrocardiogram (ECG) monitoring that exhibited relatively low impedance and maintained stable electrical resistance even under stretching. Liang et al. [354] developed a conductive and transparent hydrogel crosslinked with polypyrrole-modified microgels, suitable for neural signal recording.

### 5.5. Magnetically Sensitive Hydrogel-Based Sensors

**Figure 17:**
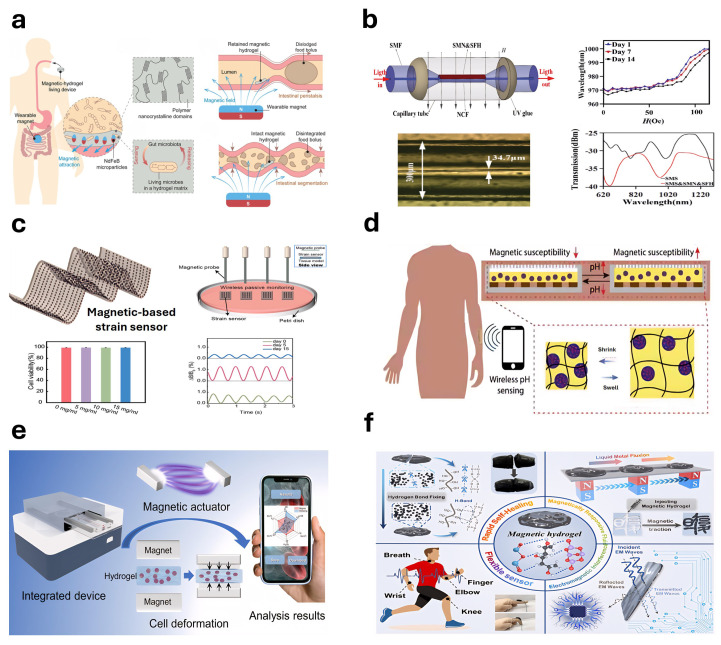
Magnetic hydrogel-based sensors: (**a**) ingestible magnetic hydrogel carriers used as position sensors (reprinted with permission from [355]); (**b**) optical fiber sensors based on magnetic hydrogel (reprinted with permission from [356]); (**c**) magnetic gel strain sensor films (reprinted with permission from [357]); (**d**) wireless sensing chemical sensors based on pH-sensitive hydrogels (reprinted with permission from [359]); (**e**) biosensors based on magnetic hydrogel actuators (reprinted with permission from [360]); (**f**) intelligent feedback sensors based on magnetic liquid metal composite hydrogels (reprinted with permission from [361]).

Magnetic hydrogel-based sensors respond to environmental stimuli by altering their physical states and converting these changes into electrical signals, thereby enabling precise environmental monitoring. Liu et al. [355] investigated an ingestible magnetic hydrogel carrier functioning as a position-localizing sensor designed to deliver diagnostic microorganisms to specific intestinal sites. Localization and retention were achieved through external magnets positioned on the abdominal skin, facilitating health monitoring and sustained drug release (Figure 17a). Ning et al. [356] developed an optical fiber sensor utilizing a mixture of streptavidin-functionalized magnetic nanoparticles, exhibiting a maximum sensitivity of 673.53 pm/Oe and an average sensitivity of 263 pm/Oe within a magnetic field intensity ranging from 0 to 115 Oe (Figure 17b). Zhang et al. [357] introduced a flexible and biocompatible magnetic strain sensor film composed of gelatin methacryloyl (GelMA) integrated with Fe_3_O_4_, capable of stably monitoring micro-strains as low as 50 µm (Figure 17c). Kessler et al. [358] explored a hydrogel sensor system containing high concentrations of magnetic particles, employing the Hall effect to measure the gel’s degree of swelling. Park et al. [359] utilized changes in ferrogel susceptibility to implement wireless chemical sensing through pH-induced hydrogel shrinking and swelling effects (Figure 17d). Yu et al. [360] proposed a biosensor based on magnetic actuation principles, utilizing magnetically actuated hydrogel stamping to assess blood storage quality (Figure 17e). Zhao et al. [361] investigated a self-healing, stretchable, and stimulus-responsive magnetic liquid metal composite hydrogel suitable for smart feedback sensors and high-performance electromagnetic shielding in wearable devices (Figure 17f). Heidarian et al. [362] employed a straightforward fabrication method to produce an elastic, magnetically responsive, and ionically conductive ferrogel for strain sensing applications. Cao et al. [363] reported a highly stretchable and magnetically responsive conductive hydrogel specifically developed for strain sensor applications.

### 5.6. Thermosensitive Hydrogel-Based Sensors

**Figure 18:**
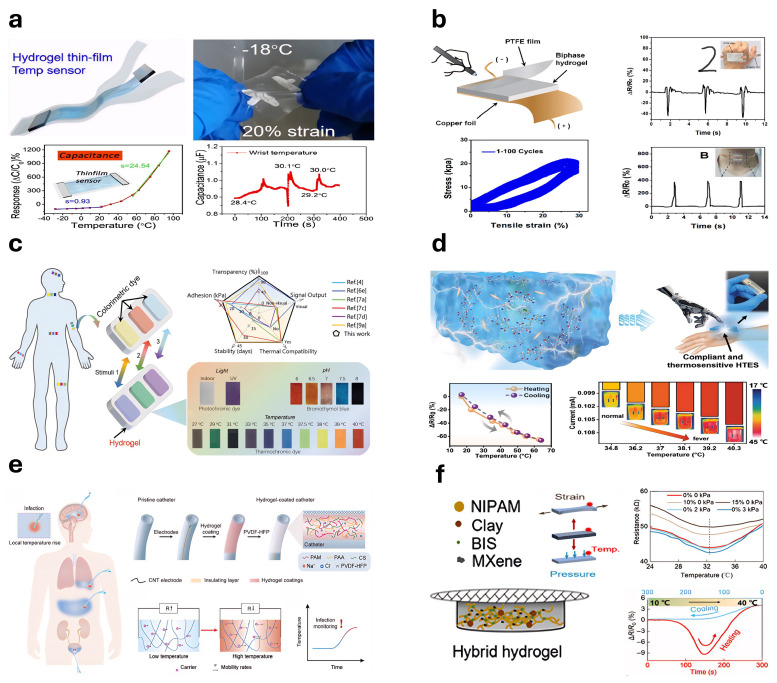
Thermosensitive hydrogel-based sensors: (**a**) high-performance hydrogel temperature sensor film (reprinted with permission from [365]); (**b**) microphase separation characteristics in thermosensitive biphasic hydrogels for high-performance sensors (reprinted with permission from [367]); (**c**) thermochromic hydrogels as wearable epidermal sensors (reprinted with permission from [368]); (**d**) thermistor epidermal sensors (reprinted with permission from [369]); (**e**) real-time temperature sensing hydrogel coating sensors for medical catheters (reprinted with permission from [370]); (**f**) hybrid MXene/clay/PNIPAM (MCP) hydrogel temperature sensors (reprinted with permission from [371]).

Thermo-hydrogel-based sensors are devices fabricated from hydrogel materials designed to measure and monitor temperature by converting temperature variations into usable output signals [364]. Wu et al. [365,366] reported a high-performance hydrogel temperature sensor with an ultrathin profile. This sensor exhibited significantly higher thermal sensitivity in capacitive mode than traditional conductive/resistive sensors, making it suitable for monitoring human motion and skin temperature (Figure 18a). Gao et al. [367] utilized microphase separation in biphasic hydrogels, achieving various microphase structures by adjusting the oil-to-water ratio and temperature conditions (Figure 18b). Han et al. [368] developed a thermochromic hydrogel serving as a wearable epidermal sensor with excellent thermal compatibility and long-term stability, demonstrating sustained performance even under extreme temperatures ranging from −20 °C to 60 °C and prolonged storage conditions of up to 45 days (Figure 18c). Hao et al. [369] introduced a thermistor-based epidermal sensor consisting of a thermally responsive and self-adhesive PEST layer with silver electrodes applied via surface spraying. The design leveraged tannic acid-modified cellulose nanocrystals to balance thermal sensitivity and adhesion properties (Figure 18d). Li et al. [370] described a real-time temperature-responsive hydrogel coating for medical catheters, combining high-temperature sensitivity, stability, and low inflammatory risks to optimize in vivo monitoring performance (Figure 18e). Wu et al. [371] fabricated a temperature sensor using hybrid MXene/clay/PNIPAM hydrogels exhibiting high stretchability and thermal stability. At temperatures exceeding 32 °C, its 3D network transformed into a 2D planar structure, significantly altering its electrical resistance (Figure 18f). Seo et al. [372] developed micropillar array temperature sensors using NIPAAm-based hydrogels reinforced with gold nanoparticles. Lastly, Ryu et al. [373] harnessed thermoresponsive hydrogels by integrating photonic crystals, entanglement-induced strategies, antifreeze additives, and multivalent metal ions to enhance their properties comprehensively, enabling practical applications in smart windows.

### 5.7. Gas-Sensitive Hydrogel-Based Sensors

**Figure 19:**
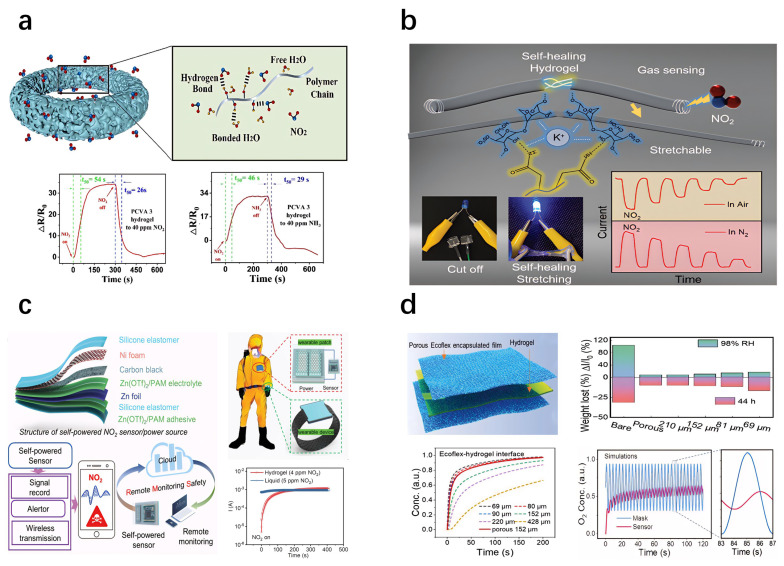
Hydrogel-based gas sensors: (**a**) Hydrogel gas (e.g., ${\mathrm{NO}}_{2}$, ${\mathrm{NH}}_{3}$) sensors (reprinted with permission from [375]); (**b**) Stretchable, self-healing, transparent ${\mathrm{NO}}_{2}$ gas sensor (reprinted with permission from [376]); (**c**) A novel self-powered flexible ${\mathrm{NO}}_{2}$ sensor based on zinc trifluoromethanesulfonate (${\mathrm{Zn}(\mathrm{OTf})}_{2}$/polyacrylamide (PAM) hydrogel–carbon structure (reprinted with permission from [377]); (**d**) Hydrogel-based oxygen sensor (reprinted with permission from [378]).

Gas-sensitive hydrogel-based sensors are advanced devices utilizing hydrogels capable of detecting and responding to specific gaseous analytes through changes in physical or chemical properties. Puttasakul et al. [374] proposed a polyacrylamide hydrogel in electrochemical gas sensors specifically designed for explosive detection. The sensor demonstrated a detection capability of one hour, with electrodes remaining stable in a Fe^2+^/Fe^3+^ solution for merely one week. Increased working temperatures significantly shortened the hydrogel’s operational lifetime; for example, at 32 °C, the sensor remained functional for one hour, whereas at 40 °C, the operational time decreased drastically to only six minutes. Zhi et al. [375] developed a rapid, reversible, and reusable gas sensor based on a supramolecular hydrogel, capable of autonomous response to specific gases such as NO_2_ and NH_3_. The responsive behavior of this hydrogel is driven by three synergistically interacting supramolecular mechanisms, hydrogen bonding, molecular crystallization, and electrostatic interactions, involving hydroxyl groups, crystalline polyvinyl alcohol, and polyionic liquids within the hydrogel network (Figure 19a). Wu et al. [376] introduced a stretchable, self-healing, and transparent NO_2_ gas sensor utilizing calcium chloride-reinforced, salt-infused ionic conductive hydrogels, enhancing sensing performance, stability, and conductivity. The self-healing property ensures the sensor maintains functionality even after mechanical damage (Figure 19b). Further research led to the fabrication of a novel self-powered flexible NO_2_ sensor based on a zinc trifluoromethane sulfonate (Zn(OTf)_2_)/polyacrylamide hydrogel–carbon structure for wireless detection of excessive NO_2_ gas. This sensor exhibits exceptionally high sensitivity and humidity immunity [377](Figure 19c). In extended studies, an oxygen sensor that is room-temperature sensitive, breathable, waterproof, and stretchable has been developed utilizing PAM/CARR hydrogels encapsulated by Ecoflex elastomers (Figure 19d). This sensor employs benzophenone-enhanced chemical crosslinking to maintain structural integrity in wearable applications, even under deformation conditions [378].

### 5.8. Photosensitive Hydrogel-Based Sensors

**Figure 20:**
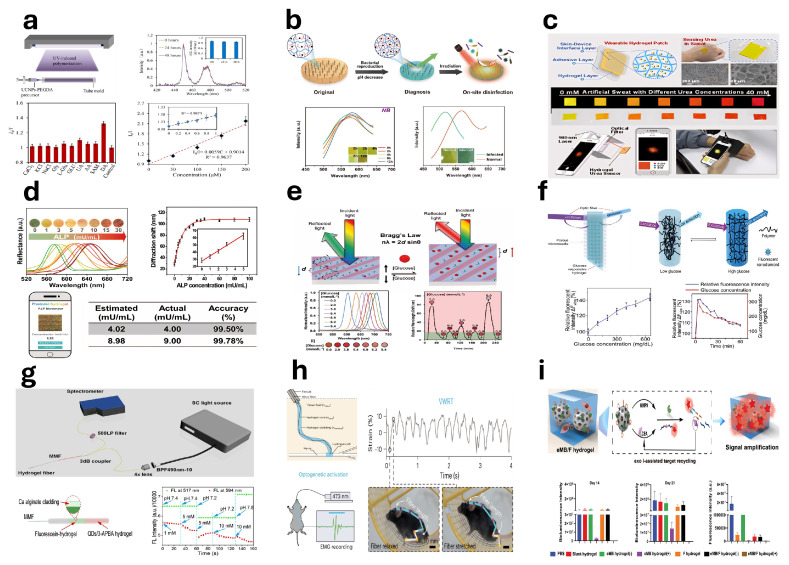
Hydrogel optical sensors: (**a**) dopamine detection hydrogel optical sensor (reprinted with permission from [379]); (**b**) pH-responsive photonic hydrogel sensor (reprinted with permission from [380]); (**c**) wearable sensor patch based on upconversion nanoparticles (reprinted with permission from [381]); (**d**) supramolecular photonic hydrogel biosensor for alkaline phosphatase (ALP) detection (reprinted with permission from [382]); (**e**) holographic hydrogel glucose sensor (reprinted with permission from [383]); (**f**) porous microneedle CGM sensor containing fluorescent nanodiamond boron hydrogel (reprinted with permission from [384]); (**g**) hydrogel optical fiber sensing functionalized with fluorescein derivatives and CdTe quantum dots/3-APBA (reprinted with permission from [385]); (**h**) hydrogel optical fiber sensor for optogenetic therapy (reprinted with permission from [386]); (**i**) hydrogel sensor integrating eMB nanoprobe and fibroblasts (reprinted with permission from [387]).

Hydrogel-based optical sensors are devices that detect the presence of specific parameters or substances in the environment by exploiting changes in the physical and chemical properties of hydrogels. Zhou et al. [379] reported a flexible, biocompatible luminescent hydrogel optical sensor employing hydrogel fibers and upconversion nanoparticles, which enabled simple and compact dopamine detection (Figure 20a). Xie et al. [380] developed a pH-responsive photonic hydrogel sensor that allows for the direct visual detection of bacteria via pH-induced color changes. At the same time, its photothermal conversion capability effectively inactivates bacteria under near-infrared illumination (Figure 20b). Hu et al. [381] designed a wearable patch incorporating upconversion nanoparticles and upconversion fluorescence probes embedded in a PAM hydrogel, which, through near-infrared excitation and the inner filter effect, enabled multiplexed chromatic response monitoring of urea levels in body fluids on a portable platform (Figure 20c). Lu et al. [382] developed an easily fabricated supramolecular photonic hydrogel biosensor tailored explicitly for the highly sensitive detection of alkaline phosphatase (Figure 20d). Davies et al. [383] fabricated a stable, nanoparticle-free holographic hydrogel glucose sensor using a single-flash ultraviolet dual-photopolymerization technique (Figure 20e). Zhang et al. [384] developed a porous microneedle continuous glucose monitoring (CGM) sensor incorporating a fluorescent nanodiamond boron-doped hydrogel based on optical principles for continuous blood glucose monitoring (Figure 20f). Li et al. [385] introduced a hydrogel optical fiber sensor functionalized with fluorescein derivatives and CdTe quantum dots/3-APBA, which achieved the simultaneous continuous monitoring of pH and glucose levels (Figure 20g). Liu et al. [386] developed a soft, stretchable, and fatigue-resistant hydrogel optical fiber intended for use in optogenetic therapy (Figure 20h). Geng et al. [387] devised a hydrogel sensor with excellent enzyme resistance by integrating eMB nanoprobes with fibroblasts; under visual guidance, the sensor rapidly performs photothermal ablation via polydopamine nanoprobes, effectively addressing residual microtumors (Figure 20i). Li et al. [388] presented a fluorescent intensity ratio fiber-optic temperature sensor by dispersing two colors of polystyrene microspheres in a CMC-Na hydrogel, exploiting the differences in the fluorescent emission wavelengths and temperature sensitivities of the microspheres to achieve continuous temperature monitoring.

### 5.9. Multifunctional Hydrogel-Based Sensors

A multimodal sensor is a device that integrates two or more distinct sensing functions, enabling the simultaneous detection and measurement of multiple physical, chemical, or biological parameters. Liu et al. [389] employed the rapid self-assembly of photonic crystal hydrogels to construct a wearable sensor capable of monitoring both strain and temperature. This sensor exhibits excellent mechanical performance, responds quickly to external stimuli, and achieves precise monitoring via visual color changes and electrical signal output. Pang et al. [390] reported a temperature-responsive ionic conductive hydrogel suitable for strain and temperature sensing. In a subsequent study, they prepared a PNIPAAm-based nanogel-crosslinked PSBMA network, demonstrating sensitive temperature-to-electrical signal transduction over 30–45 °C [391]. Zhao et al. [392] used a one-pot mixing method of SH, gelatin, and borax to obtain a DN SBG conductive hydrogel that supports dual sensing of strain and temperature. Xu et al. [393] introduced an ionic liquid/EG/water ternary solvent system to enhance the compatibility between starch and PVA, and through freeze–thaw combined with multiple hydrogen bonds, fabricated an SAEP ionic conductive hydrogel suitable for strain, pressure, and humidity sensing. Ge et al. [394] developed an electronic skin via dynamically reinforced RS-Ag interactions coupled with covalent crosslinking, endowing the material with self-adhesion, self-healing, biodegradability, and outstanding mechanical properties alongside high-sensitivity detection of temperature and strain.

Qu et al. [395] incorporated poly(ionic liquids) into PAA/PAM hydrogels to prepare intelligent multi-network hydrogels with both strain and temperature sensing capabilities. In another work, Qu et al. [396] integrated MXene and quaternized chitosan into a binary polymer chain, creating a hydrogel sensor with ultrahigh sensitivity to solvent and temperature. Yin et al. [397] presented a conductive hydrogel composed of sodium carboxymethyl cellulose (SCMC) and MXene, offering dual strain and temperature sensing functionality. Fu et al. [398] established a novel hydrogel with excellent mechanical properties and conductivity based on a triple physical crosslinking structure, exhibiting high sensitivity to strain, pressure, and temperature. Qin et al. [399] reported an ionic conductive, anti-drying, and flexible organic hydrogel suitable for pressure and gas sensing. Sun et al. [400] developed a multifunctional hydrogel sensor by combining PVA and gelatin biopolymers, simultaneously detecting pressure/strain, humidity, temperature, and human motion.

Wang et al. [401] fabricated a composite hydrogel based on biomass-derived chitosan quaternary salts and liquid metal, employing it in a dual-parameter smart wearable sensor for temperature and stress. Cheng et al. [402] introduced a multimodal solid soft sensor by integrating hydrogel with silicone, enabling simultaneous detection of deformation and temperature. Feng et al. [403] designed a conductive gel featuring enhanced toughness, self-healing, and self-adhesion; using this gel, they built a dual-response sensor capable of sensing both strain and temperature. Zhou et al. [404] constructed a double-network hydrogel with triple noncovalent crosslinking, yielding a highly sensitive pressure and strain sensor. Gao et al. [405] applied a one-pot strategy to develop a flexible cellulose-based conductive hydrogel suitable for strain and temperature sensing. Zhu et al. [406] created a dual-peak hydrogel sensor by locally coating PEDOT:PSS to form separate regions for strain and temperature detection, thereby achieving high sensitivity, a broad detection range and signal decoupling for precise measurement and enhanced cycling stability. He et al. [407] prepared a conductive hydrogel by combining PNIPAM microgels with gallium-based liquid metal, delivering excellent mechanical performance, temperature responsiveness, and electrical conductivity; it demonstrates high strain sensitivity (gauge factor up to 5.45) and a wide temperature detection range (20–70 °C). Zhang et al. [408] reported an intrinsically anti-freezing, microphase-separated hydrogel that confines water molecules within nanochannels and increases the proportion of bound water, thereby attaining superior freeze tolerance, high mechanical strength, and optical transparency for use in strain and pressure sensors. Lastly, Chen et al. [409] developed a wearable hydrogel-based sweat sensor that combines surface-enhanced Raman spectroscopy (SERS) with artificial intelligence algorithms to enable the noninvasive, high-frequency monitoring of lung cancer treatment efficacy.

### 5.10. Perspectives

Hydrogel-based sensors demonstrate broad potential for diverse applications due to their unique flexibility, biocompatibility, and multifunctionality. Significant advances have been made in hydrogel-based strain and pressure sensors and sensors sensitive to humidity, conductivity, magnetic fields, heat, gases, photosensitivity, and other sensing modalities. These technological innovations expand traditional sensor application scenarios and open new developmental avenues in cutting-edge fields such as intelligent wearable devices, biomedical instrumentation, and robotics. However, similar to the future development trajectory of hydrogel-based actuators, which relies heavily on integrating actuation with sensing and control, the evolution of hydrogel-based sensors must increasingly align closely with specific application contexts. Future developments should prioritize achieving a higher degree of integration between hydrogel-based sensing technology and practical application requirements. For instance, in humanoid robotics, constructing flexible interfaces with hydrogel sensors can enable safer and more natural interactions between robots and humans. In medical applications, integrating hydrogel sensors into flexible surgical instrument interfaces could significantly enhance the safety and precision of surgical procedures. Thus, advancing hydrogel-based sensor technology in the future requires transitioning further from fundamental research toward deep integration with concrete application scenarios, facilitating technology deployment in practical contexts and generating new industrial value.

## 6. Discussion

This review comprehensively examines the progress in hydrogel-based continuum soft robots, encompassing their applications, fabrication methods, actuators, and sensing. It highlights hydrogels’ distinctive advantages in enabling flexible structures and adaptive responses and underscores their pivotal role in high-precision, multimodal sensing from the perspectives of materials, design, and applications. Compared with traditional robots that rely on rigid structures, hydrogels present novel possibilities for simulating biological skin, facilitating efficient sensing, and advancing real-time control technologies, offering new design paradigms for functional integration and cross-disciplinary applications of soft robots. Although the current research still faces challenges related to the long-term stability of materials, environmental adaptability, and integrated manufacturing processes, these preliminary achievements have laid a solid foundation for future deployment in complex scenarios such as medical, industrial, and agricultural settings, while also providing valuable insights for interdisciplinary technological integration.

Over years of intensive investigation in biomedicine, chemistry, and materials science, hydrogel materials have evolved into various systems, exhibiting exceptional physicochemical properties. Many hydrogels possess high biocompatibility, remarkable mechanical flexibility, and notable attributes in self-healing and responsive regulation (e.g., temperature, pH, and light sensitivity). Such properties confer irreplaceable advantages in drug delivery, tissue engineering, and innovative materials. However, despite the continuous breakthroughs in individual hydrogel performance in laboratory settings, the cross-disciplinary integration of these outstanding features into functional continuum soft robot systems remains challenging. First, although hydrogels often demonstrate excellent indicators—such as response speed, energy conversion efficiency, and long-term stability—when investigated as single materials, these properties must be organically coupled with actuators, sensing, control, and energy management modules in robotic systems. The current research focuses on material optimization but lacks a deeper exploration of holistic system integration and engineering implementation. Second, real-world applications of soft robots demand high robustness and dynamic adaptability in complex environments, whereas conventional hydrogel studies typically occur under relatively ideal laboratory conditions. Ensuring that hydrogels retain functionality and durability under realistic operating conditions thus remains an urgent challenge.

Hydrogel-based continuum soft robots exhibit tremendous application potential in medical assistance, precision operations, industrial inspection, and agricultural monitoring. For instance, they enable the precise manipulation of soft tissues in minimally invasive surgeries and provide dynamic feedback for rehabilitation training. In industrial and agricultural settings, they facilitate real-time equipment fault detection and the accurate acquisition of environmental parameters via high-precision sensing. Nonetheless, their broader deployment faces formidable challenges. On one hand, there is an urgent need to enhance hydrogels’ long-term stability and self-healing capabilities in complex environments characterized by fluctuating temperature and humidity as well as chemical corrosion. On the other hand, different fields present significantly diverse integration requirements for sensors, actuators, and control systems. Consequently, transitioning from laboratory prototypes to customized commercial systems involves not only addressing the real-time control accuracy and response speed issues arising from nonlinear dynamics but also establishing robust safety monitoring mechanisms to ensure reliability in human–machine interactions.

Future research should concentrate on integrating hydrogels with AI technologies to expand and empower the rapid commercial deployment of conventional rigid robots. By leveraging the exceptional flexibility, self-healing properties, and multimodal responsiveness of hydrogels, together with big data and AI, traditional rigid robots can achieve high-precision, real-time sensing functionalities while demonstrating enhanced safety and adaptability in complex application scenarios such as medical, rehabilitation, and household settings. The commercialization of this breakthrough technology will infuse substantial research and development funding into the field, accelerating technology transfer and industrialization. Meanwhile, advances in and applications of hydrogel technologies will establish a solid foundation for the evolution of hydrogel-based continuum soft robots. Through deep integration with robotics design, control theory, and systems engineering, hydrogel-enabled solutions will not only bridge the gap from theory to practice but also drive fundamental breakthroughs in robotic safety, intelligence, and environmental adaptability. Furthermore, from a top-level perspective, it is essential to create highly collaborative, cross-disciplinary research and development platforms that dismantle barriers among disciplines, thereby achieving a two-way synergy between technology and commercialization and propelling the robotics field into a new era of widespread application.

## 7. Conclusions

This review systematically summarizes recent progress in several key aspects of hydrogel-based continuum soft robots, including their applications, fabrication methods, actuation mechanisms, and sensing capabilities. Its primary objective is to draw the attention of researchers in both hydrogel science and continuum robotics, thereby promoting deeper integration between hydrogel materials and robotic technologies. Leveraging artificial intelligence to enhance hydrogel materials and constructing diverse continuum soft robots is expected to open up new avenues for applications such as drug delivery and targeted release. Meanwhile, inspired by the natural combination of hydrogels and rigid skeletal structures in biological systems, hydrogel-based soft robots exhibit marked advantages in flexibility and safety compared to traditional rigid robots. In practical scenarios, the complex structures of conventional robots and the inherent risk of system failures often limit their ability to interact directly with humans. In contrast, hydrogel-enabled robots more closely align with human activity patterns, providing safer and more adaptable services. In summary, hydrogel-based continuum soft robots not only introduce a novel research direction in robotics but also offer new possibilities for overcoming a range of challenges in biomedicine and human–robot interaction. We hope that this review will inspire more interdisciplinary collaborations and collectively drive further advancements in this rapidly evolving field.

## Figures and Tables

**Table 1:** Industrial application of hydrogel-based continuum soft robots.

Research Area	Research Direction	Hydrogel	Ref.
Flexible Electronics	Ionic hydrogel sensors	Collagen-PAAm	[53]
	Enhanced mechanical properties	Metal-coordination HGs	[54]
Environmental Applications	Solar-driven water purification	LSAG	[55]
	Pollutant adsorption mechanisms	Double-network HGs	[57]
Soft Robotics	Extreme-environment robotics	Multifunctional ionic HGs	[56]
	Gradient-responsive robotics	Gradient-structured HGs	[58]
	Medical simulation robotics	Carbon-nanomaterial HGs	[59]
	Fire-resistant robotic skins	MMT-biocompatible HGs	[60]
Microfabrication and Security	Micro/nano photonics fabrication	Photonic HGs	[51]
	3D encryption/decryption	pH-responsive degradable HGs	[61,62]

**Table 2:** Invivo and in vitro medical applications of hydrogel-based continuum soft robots.

Research Methods	Indications	Hydrogel	Ref.
Vivo	Infected wound	PN-AP	[63]
	Acutely injured retina	Cur@PDA@GelCA	[68]
	Wounds in infants and diabetics	PGA-GelMA	[76]
	Gastrointestinal Treatment	e-GLUE	[77]
	Fostering bone regeneration	O2-PSSG	[69]
	Contact lenses	PVA	[73]
	Tumor treatment	CR–PLD–Fe_3_O_4_–Ag	[78]
	Oral insulin delivery	A-C-DNA	[79]
	Oral cavity wound	PAM-G-CS	[80]
	Hemostasis and Wound Sealing	RAAS	[81]
	Magnetic controlled drug release	SA-NHS@CS	[71]
	Active target delivery	NEs@EM@nanogels	[72]
Vitro	Sensor/Intelligent manipulator	Nacl@PAAm	[82]
	Sensor/Continuum robots	Na-Alg@PDMS	[83]

**Table 3:** Applications of hydrogel-based continuum soft robots in agriculture, food science, and cosmetics.

Field	Application	Hydrogel	Ref.
Agriculture	Fertilizer and water management	Superabsorbent hydrogel	[95]
	Pesticide detection	Wearable hydrogel sensor	[96]
	Urban agriculture	Hydrogel system	[97]
	Atmospheric water harvesting	Biopolymer hydrogel	[98]
	Drought resistance	Functional hydrogel	[99]
	Fungicide delivery	Polysaccharide supramolecular hydrogel	[100]
	Soil moisture retention	Lignin-based hydrogel	[101]
	Nitrate storage	PNIPAM hydrogel	[102]
Food Science	Food processing	Pectin hydrogel	[115]
	Bioactive delivery	Chitosan hydrogel	[103]
	Bioactive release	Starch hydrogel	[104]
	Structural diversity	Hydrogel crosslinking (Review)	[116]
	Food application	Peptide hydrogels (Review)	[117]
	Food safety	Functional hydrogels (Review)	[105,106]
	Food packaging enhancement	Edible polymer hydrogel	[107]
	Sustainable packaging	Gelatin-based hydrogel	[108]
	Food preservation	Carboxymethyl hydrogel film	[109]
	pH-responsive packaging	Polysaccharide hydrogel	[110]
Cosmetics	Formulation development	Chitosan, hyaluronic acid, alginate	[118]
	Dermatology	Multifunctional hydrogel	[111]
	Moisturizer	Hyaluronic–silicon hydrogel	[112]
	Anti-aging	Fullerene-polysaccharide hydrogel	[113]
	Safety testing	Hyaluronic acid hydrogel film	[119]
	Exfoliating product	Enzyme-based hydrogel	[120]
	Moisturizing additives	Green tea nanoparticle hydrogel	[121]
	Sunscreen	Yeast–gelatin hydrogel	[114]

**Table 4:** Literature produced by different crosslinking methods.

Crosslinking Type	Crosslinking Mechanism	Reaction Conditions	Ref.
Chemical	Radical Polymerization	Photoinitiator	[129]
	Dynamic covalent (1,2-dithiolane)	Photoinduced	[130]
	Enzyme(TGase)	Temperature/pH	[131]
	UV	2,2-Dimethoxy-2-phenylacetophenone	[132]
Physical	Ionic	CaCO_3_	[133]
	Hydrogen Bonds	HCl	[134]
	Hydrogen Bonds	Dimethyl Sulfoxid/Hydrogen-bond Acceptor	[135]
	Ionic/Hydrogen Bonds	2-Amino-4-hydroxy-6-methylpyrimidine/Dimethyl Sulfoxid	[136]
	Ionic/Hydrogen Bonds	H_2_SO_4_	[137]
	Collaborative Hydrogen Bonds	2-methyl-2-benzyloxy carbonyl propylene carbonate	[138]
Both	Free Radical/Hydrogen Bond	sGOa/mGOa	[139]
	Enzymatically Catalyzed Crosslinking	Horseradish Peroxidase/H_2_O_2_	[140]
	Ionic/UV	KCl/I2959	[141]
	Freezing/UV	Irgacure 2959	[142]
	Ionic/Enzyme(TG)	TG/Gacl	[143]
	Hydrogen Bonds/UV	HCL/I2959	[144]

## Data Availability

Data are contained within the article.
